# CED-RTDETR: a contour-aware evidence-guided decoupled network for rice leaf disease detection

**DOI:** 10.3389/fpls.2026.1891279

**Published:** 2026-08-19

**Authors:** He Liu, Changlin Li, Yuhang Cao, Jiamu Wang, Chunxi Zhao

**Affiliations:** 1 College of Engineering and Technology, Jilin Agricultural University, Changchun, China; 2 College of Information Technology, Jilin Agricultural University, Changchun, China; 3 Information Center, Jilin Agricultural University, Changchun, China

**Keywords:** contour enhancement, deformable attention, multi-scale evidence fusion, rice disease detection, RT-DETRv2, small object detection

## Abstract

To address the challenges posed by large lesion-scale variations, weak boundary cues, and high inter-class similarity in rice leaf disease detection, this study proposes an improved RT-DETRv2-R50-based rice disease detection model, termed CED-RTDETR. First, a Lightweight Contour-Guided Aggregation Backbone (LCGA-Backbone) is constructed. In the PResNet residual blocks, an InceptionDWConv2d-based direction-aware depthwise separable spatial mixing strategy is introduced to capture local, horizontal, and vertical lesion texture patterns with low computational overhead. Meanwhile, a Contour-guided Efficient Global Aggregation Block (CEGA Block) is embedded after the outputs of the C3, C4, and C5 stages. Through contour-difference enhancement, channel shuffle, group-wise efficient global aggregation, and bottleneck channel mixing, the proposed block strengthens the representation of the boundaries of small lesions, weak textures, and contextual semantics. Second, a Multi-scale Evidence Interaction Fusion Neck (MEIF-Neck) is used to perform multi-scale evidence interaction and salient-region competition after cross-scale feature concatenation. A lightweight feature reconstruction process is further implemented using Spatial-Evidence RepNCSPELAN (SE-RepNCSPELAN), which is developed from a YOLO-style feature fusion structure. Finally, a Direction-Amplitude Decoupled Deformable Attention (DAD-DA) mechanism is introduced to decompose sampling offsets into direction rotation residuals and radius gains while incorporating an aspect-ratio-aware geometric compression-restoration strategy, thereby improving the geometric stability of sampling locations during the decoding stage. Experimental results on the constructed rice leaf disease dataset show that CED-RTDETR achieves AP, AP50, and AP75 values of 29.5%, 75.2%, and 17.6%, respectively, outperforming RT-DETRv2-R50 by 4.1, 4.9, and 3.8 percentage points. To further evaluate the model on an additional public benchmark dataset, experiments were also conducted on the public Rice Disease Dataset. On this dataset, CED-RTDETR achieves AP, AP50, and AP75 values of 34.1%, 77.1%, and 23.2%, respectively, improving upon RT-DETRv2-R50 by 4.3, 5.6, and 3.4 percentage points. These results indicate that the proposed method achieves consistent overall performance improvements on both the constructed dataset and the public benchmark dataset.

## 1 Introduction

In recent years, deep learning has greatly promoted the application of computer vision in smart agriculture. With the development of unmanned aerial vehicles, field imaging platforms, and embedded vision devices, agricultural image analysis has gradually expanded from controlled image recognition to more complex scenarios involving field monitoring, crop growth assessment, weed–crop discrimination, fruit maturity estimation, and plant disease detection. For example, UAV-assisted blueberry maturity monitoring has combined aerial image acquisition with Mamba-based computer vision to improve maturity assessment under field imaging conditions (Zhao et al., 2025a), while UAV-based weed and crop segmentation has shown the importance of accurate crop–background separation for precision agriculture (Tao et al., 2025). These studies indicate that agricultural vision models are increasingly required to handle variable imaging conditions, complex backgrounds, and fine-grained target differences.

At the same time, recent agricultural and ecological detection studies have further shown that model structures need to be adjusted according to task-specific visual characteristics. For underwater or aquatic monitoring, Mamba-based super-resolution and semi-supervised YOLOv10 have been used to improve freshwater mussel detection from acoustic video data (Zhao et al., 2025c), while water-aware YOLO-based detection has been explored for floating plastic debris under complex water-surface backgrounds (Liu et al., 2026). In UAV-based plant monitoring, Succulent-YOLO combines CLIP-based YOLOv10 and Mamba-related visual enhancement for succulent farmland monitoring (Li et al., 2025), and ROSE-YOLO and Rose-Mamba-YOLO improve rose detection by considering image degradation, scale variation, occlusion, and complex greenhouse backgrounds (You et al., 2025; Zhao et al., 2025b). These works suggest that lightweight inference, multi-scale feature fusion, contextual modeling, and background-interference suppression are key issues when deep learning models are applied to complex agricultural images.

Rice leaf disease detection is a typical and challenging task in this context. It aims to automatically identify disease categories and localize visible lesion regions, thereby supporting disease image analysis and lesion-level assessment. Diseases such as bacterial leaf blight, rice blast, and brown spot can impair photosynthetic activity and adversely affect rice yield. Traditional manual inspection is often inefficient, subjective, and difficult to adapt to complex field environments. Therefore, automated detection methods are needed to improve the efficiency and consistency of rice disease image analysis.

Object detection can simultaneously perform category recognition and lesion localization. Existing object detection methods can be broadly divided into two-stage detectors and one-stage detectors. Two-stage detectors, such as R-CNN, Fast R-CNN, Faster R-CNN, and Cascade R-CNN, usually achieve high detection accuracy, but their detection pipelines are relatively complex (Girshick et al., 2014; Girshick, 2015; Ren et al., 2015; Cai and Vasconcelos, 2018). In contrast, one-stage detectors, such as SSD, RetinaNet, and the YOLO series, have simpler architectures and higher inference efficiency, and have therefore been widely used in real-time detection tasks (Liu et al., 2016; Lin et al., 2017b; Jocher and Qiu, 2024; Wang et al., 2024; Jocher et al., 2026). The above agricultural studies also show that simply applying a general detector is often insufficient for complex agricultural scenes. Instead, model improvements typically target specific visual challenges, such as small targets, occlusion, low-resolution imagery, background interference, and real-time inference requirements.

In the field of rice disease detection, the YOLO series has also been extensively investigated. For example, G-YOLO improves rice leaf disease detection by introducing a lightweight detection head and multi-scale spatial pyramid pooling (Gan et al., 2025). TLI-YOLO focuses on lightweight modifications to YOLOv8 and mobile compatibility for rice disease detection (Li et al., 2025). RLDD-YOLOv11n enhances rice leaf disease detection in complex scenarios by improving neck feature representation (Fang et al., 2025). These studies suggest that lightweight structures, multi-scale feature fusion, and attention enhancement are effective directions for improving rice disease detection models. However, most existing improvements still mainly focus on general feature enhancement or efficient detection, while the specific visual characteristics of rice leaf lesions, such as weak contours, stripe-like symptoms, and small scattered spots, require further consideration.

Rice leaf disease detection faces several specific challenges. First, brown spot lesions often appear as small and scattered spots, and their fine details are easily weakened during downsampling. Second, rice blast and brown spot may show similar color, texture, and local morphology, which can lead to inter-class confusion. Third, rice leaf images are easily affected by leaf veins, illumination variation, shadows, and cluttered backgrounds, making weak lesion boundaries difficult to distinguish. Fourth, bacterial leaf blight and some rice blast symptoms may form slender or stripe-like lesion regions along the leaf direction, which increases the difficulty of stable bounding-box localization. These characteristics require the detector to strengthen contour preservation, directional texture extraction, cross-scale lesion evidence fusion, and geometrically stable localization.

In addition to the YOLO series, DETR-based end-to-end detection frameworks provide another perspective for crop disease detection. DETR models object sets through object queries (Carion et al., 2020), while Deformable DETR improves feature aggregation efficiency by introducing multi-scale deformable attention (Zhu et al., 2021). RT-DETR further balances real-time inference and detection accuracy (Zhao et al., 2024). ADAM-DETR applies an improved RT-DETR to rice disease detection, showing that DETR-based models can be further optimized for this task (Song et al., 2025). Nevertheless, when RT-DETRv2 is directly applied to rice leaf disease detection, its backbone feature extraction, neck feature fusion, and decoder sampling mechanism are not fully suited to small, weak-boundary, and irregular lesion regions commonly observed in rice disease images (Lv et al., 2024).

Based on the above considerations, this study proposes CED-RTDETR using RT-DETRv2-R50 as the baseline, aiming to enhance rice lesion representation from the backbone, neck, and decoder. In the backbone, LCGA-Backbone introduces direction-aware spatial mixing into the PResNet residual blocks and embeds contour-guided enhancement after the C3, C4, and C5 stages, so that local spot-like lesions, stripe-like symptoms, and weak lesion boundaries can be better represented. In the neck, MEIF-Neck performs multi-scale evidence interaction and lightweight reconstruction after cross-scale feature concatenation, which helps retain small-lesion details and reduce interference from leaf veins and complex backgrounds. In the decoder, DAD-DA decouples the sampling offsets in deformable attention into direction and radius components, and constructs sampling locations through aspect-ratio-aware geometric restoration and reference-box boundary-radius constraints, improving the sampling stability for slender and irregular lesion regions. The main contributions of this study are summarized as follows:

1. An LCGA-Backbone is constructed to strengthen rice lesion feature extraction. The InceptionDWConv2d-based DIDM strategy is introduced into the PResNet residual branch to supplement local, horizontal, and vertical texture responses with low computational overhead. This facilitates the representation of spot-like brown spot lesions and stripe-like bacterial leaf blight symptoms. Meanwhile, CEGA Blocks are embedded after the C3, C4, and C5 stages to extend contour-aware feature enhancement from local texture extraction to multi-stage lesion representation, thereby improving the preservation of weak lesion boundaries and texture transition regions.
2. A MEIF-Neck is constructed to improve cross-scale lesion evidence fusion. After cross-scale feature concatenation, MDEI is introduced to extract multi-scale contextual evidence and perform response recalibration before feature reconstruction, which helps enhance small and weak-boundary lesion regions. SE-RepNCSPELAN further combines lightweight reconstruction with spatial evidence enhancement, reducing the influence of leaf veins, shadows, and background textures during feature fusion.
3. A DAD-DA mechanism is introduced to improve deformable attention sampling for irregular rice lesion localization. The original two-dimensional sampling offsets are decomposed into direction rotation residuals and radius gains. By introducing aspect-ratio-aware geometric compression-restoration and incorporating the boundary radius of the reference box, DAD-DA improves the geometric adaptability of sampling locations for slender and irregular lesion regions.
4. Main comparison experiments, ablation experiments, PR curve analysis, response visualization analysis, and detection result visualization are conducted on the constructed rice leaf disease dataset containing three categories: bacterial leaf blight, rice blast, and brown spot. In addition, experiments on the public Rice Disease Dataset are included as an additional benchmark setting. The experimental results show that the proposed method outperforms RT-DETRv2-R50 in terms of AP, AP50, and AP75 while maintaining a controllable increase in model complexity.

## 2 Related work

### 2.1 Crop disease detection methods

Early crop disease recognition methods mainly relied on manually designed color, texture, and shape features, which were combined with traditional classifiers such as support vector machines and random forests for disease identification. These methods could achieve acceptable performance under simple backgrounds and controlled imaging conditions. However, they were highly sensitive to illumination variations, leaf occlusion, and complex field backgrounds. With the development of deep learning, convolutional neural networks have become the mainstream approach for crop disease recognition and detection because of their ability to automatically extract hierarchical semantic features. A recent systematic review further showed that rice leaf disease research has gradually shifted toward deep learning-based automatic detection, while dataset diversity, model generalization, complex-scene adaptability, and efficient deployment remain important challenges (Simhadri et al., 2025).

Compared with image classification, object detection can not only identify disease categories but also localize lesion regions, making it more suitable for lesion-level image analysis and quantitative disease assessment. In recent years, YOLO-based models have been widely applied to rice disease detection owing to their real-time inference capability and ease of deployment. G-YOLO improves rice leaf disease detection performance by introducing a lightweight detection head and multi-scale spatial feature enhancement (Gan et al., 2025). TLI-YOLO focuses on mobile deployment scenarios and emphasizes the balance between detection accuracy and lightweight implementation (Li et al., 2025). RLDD-YOLOv11n improves the structure of YOLOv11n to enhance the detection performance of rice leaf diseases (Fang et al., 2025). More recent studies have continued to improve feature representation and deployment efficiency. Pyramid-YOLOv8 combines multi-attention feature fusion with an additional detection head for precise rice leaf blast detection (Cao et al., 2024). SSD-YOLO incorporates channel attention, dynamic upsampling, and shape-aware regression to improve lightweight rice leaf disease detection (Pan et al., 2025). YOLO-DP integrates cross-dimensional attention, global–local feature aggregation, and enlarged receptive fields for detecting multiple rice diseases and pests under complex conditions (Zhou and Wei, 2025). These studies indicate that rice disease detection models usually need to achieve a trade-off among accuracy, speed, model complexity, and robustness in complex environments.

In addition to YOLO-based methods, DETR-based models have gradually been introduced into crop disease detection tasks. DETR models object detection as set prediction through object queries (Carion et al., 2020). Deformable DETR improves the efficiency of multi-scale feature processing through deformable attention (Zhu et al., 2021), while RT-DETR further enhances the real-time performance of end-to-end detectors (Zhao et al., 2024). ADAM-DETR proposes an adaptive multi-scale feature fusion method for rice disease detection and indicates that subtle lesion textures, insufficient disease boundary representation, and inadequate multi-scale feature fusion in complex field environments still affect detection performance (Song et al., 2025). Therefore, rice disease detection based on RT-DETRv2 still requires further improvement in terms of backbone feature extraction, neck feature fusion, and decoder localization mechanisms (Lv et al., 2024).

Overall, existing crop disease detection methods have evolved from traditional classification approaches to real-time, lightweight, and end-to-end detection frameworks. However, most recent improvements primarily focus on general feature enhancement, multi-scale fusion, or lightweight deployment. In rice leaf disease scenarios, weak contours, directional stripe-like textures, small scattered lesions, similar disease morphology, and complex background interference remain insufficiently addressed. Therefore, coordinated enhancement of lesion contours and textures, cross-scale semantic fusion, and geometrically stable localization remains necessary.

### 2.2 Multi-scale feature fusion and attention mechanisms

Multi-scale feature fusion is an essential component of object detection. FPN integrates high-level semantic information and low-level detailed information through a top-down pathway and lateral connections (Lin et al., 2017a). PANet further introduces a bottom-up path aggregation structure based on FPN, which shortens the information transmission path from low-level localization features to high-level semantic features (Liu et al., 2018). These methods provide an important basis for the design of neck networks in detection models such as YOLO and RT-DETR.

Multi-scale fusion is particularly important for rice disease detection. Early-stage lesions are usually small in area and highly dependent on shallow texture and edge information, whereas developed lesions may form large and irregular regions that require stronger semantic representation. If only deep features are used, the fine details of small lesions can easily be weakened during downsampling. Conversely, if only shallow features are used, the model is more likely to be disturbed by background factors such as leaf veins, illumination variations, and shadows. In small-object detection tasks, YOLOv8-FSD introduces the SCA Module into the feature extraction network and designs AG-PAFPN, the MSFE Module, and the TBFF Module to enhance multi-order feature representation, multi-scale spatial information extraction, and cross-dimensional feature fusion, respectively (Guo et al., 2025). Recent RT-DETR-based agricultural detectors have further emphasized cross-scale interaction and contextual reconstruction. WMC-RTDETR combines wavelet-based multi-scale extraction, multi-scale self-attention, and context-guided feature reconstruction to improve disease detection under occlusion and complex backgrounds (Zhang et al., 2025). AF-RT-DETR introduces bidirectional cross-level interaction and dynamic receptive-field expansion to improve RT-DETRv2-based plant disease detection under scale variation, illumination changes, and leaf occlusion (Liu et al., 2026). These designs provide important references for the joint enhancement of the backbone and neck in this study.

Attention mechanisms can guide the model to focus on key regions or important channels and are commonly used to enhance responses to lesion regions. Attention modules such as SE, CBAM, and ECA are often employed for channel-wise or spatial response recalibration (Hu et al., 2018; Woo et al., 2018; Wang et al., 2020). Coordinate Attention introduces positional information into channel attention, enabling the model to obtain direction-aware and position-sensitive feature representations (Hou et al., 2021). In addition, Triplet Attention models the relationship between channels and spatial dimensions through cross-dimensional interaction, which helps enhance local details and spatial structural representation (Misra et al., 2021). These attention mechanisms provide effective references for background suppression, boundary enhancement, and key-region localization in rice lesion detection. However, most of them mainly recalibrate existing responses and do not explicitly model weak contour differences or directional lesion textures across multiple backbone stages.

In Transformer-based detectors, attention mechanisms also directly affect the object localization process in the decoder. Deformable DETR aggregates features using a small number of sampling locations around reference points, thereby reducing the computational complexity of global attention (Zhu et al., 2021). However, conventional deformable attention usually predicts two-dimensional sampling offsets directly from the query, in which direction and amplitude are coupled within the same offset vector. When lesion regions exhibit irregular contours, weak boundaries, or complex background interference, the stability of sampling locations can affect the quality of feature aggregation during decoding. Therefore, structured modeling of the sampling mechanism in deformable attention is meaningful for improving the localization capability of RT-DETRv2 in rice disease detection.

In summary, existing studies have promoted the development of disease detection models from the perspectives of multi-scale fusion, attention enhancement, and end-to-end detection. Nevertheless, three limitations remain relevant to rice leaf disease detection: insufficient explicit modeling of weak contours and directional textures in the backbone, limited interaction and salient-evidence selection before cross-scale feature reconstruction in the neck, and coupled direction–amplitude prediction in deformable sampling. Based on these considerations, this study improves RT-DETRv2 through LCGA-Backbone, MEIF-Neck, and DAD-DA, which address, respectively, weak lesion representation, cross-scale evidence fusion, and unstable localization of slender or irregular lesions.

## 3 Improved RT-DETRv2 method

### 3.1 LCGA-Backbone

**Figure 1:**
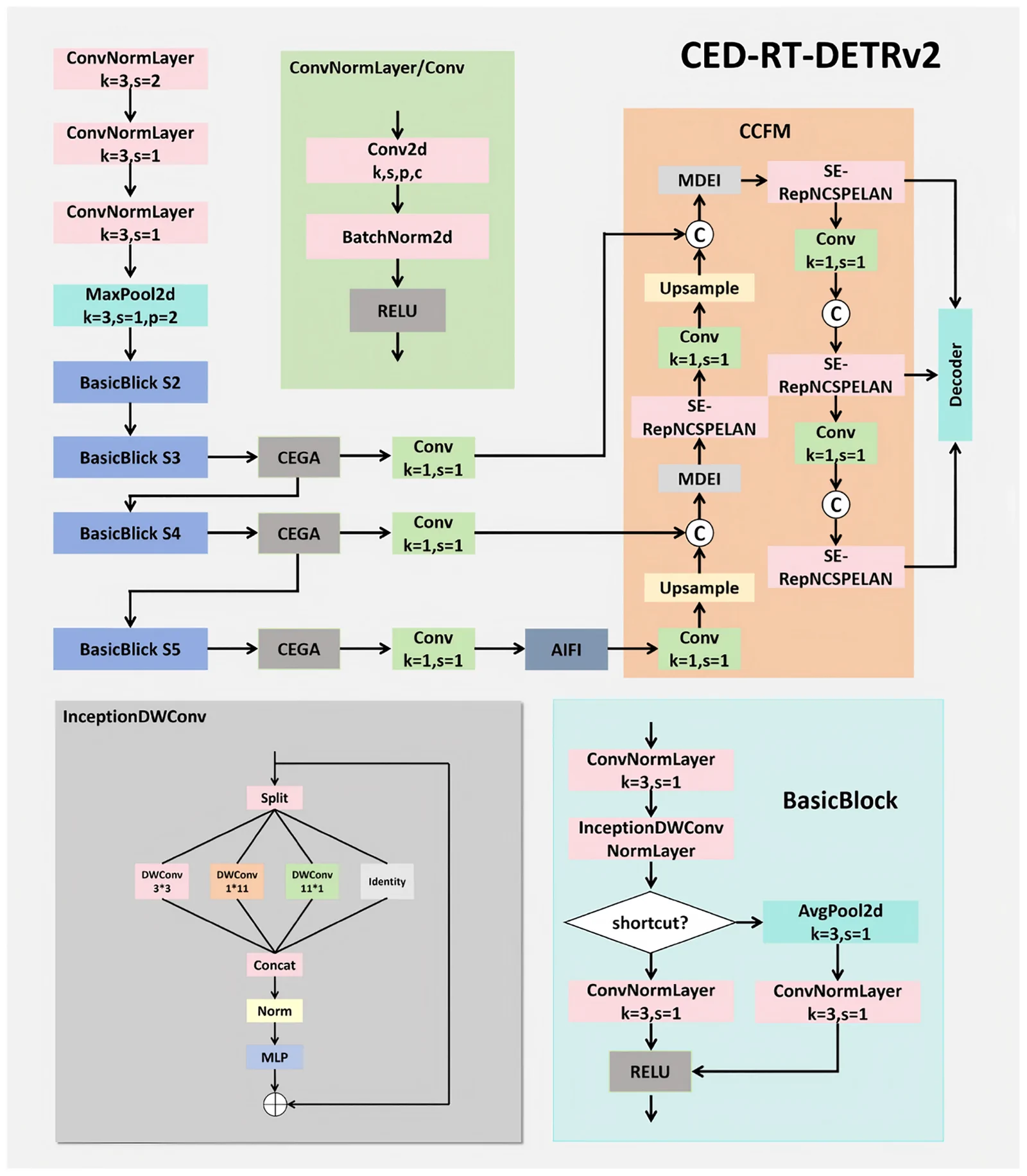
Overall architecture of the proposed CED-RTDETR network.

In this study, CED-RTDETR is constructed based on the RT-DETRv2-R50 framework. The input image is first fed into the LCGA-Backbone to extract multi-level lesion-aware features. Then, the MEIF-Neck performs cross-scale evidence interaction and lightweight fusion reconstruction. Finally, the fused features are delivered to the Transformer decoder equipped with DAD-DA for object query updating and bounding box prediction. The overall architecture of the proposed network is shown in **Figure 1**.

The original RT-DETRv2 adopts PResNet to extract multi-scale features from C3, C4, and C5, thereby providing strong general-purpose feature representations. However, in rice leaf disease detection scenarios, bacterial leaf blight, rice blast, and brown spot exhibit significant differences in scale, morphology, and directional texture. Conventional 3×3 convolutions have limited ability to distinguish local spot-like lesions, horizontally extended textures, and vertically distributed stripe-like structures. Meanwhile, as the backbone network becomes deeper and the spatial resolution of feature maps decreases, the boundary details and weak texture cues of small lesions can be attenuated. To address these problems, LCGA-Backbone enhances lesion feature representation from two aspects: low-cost directional spatial mixing within residual blocks and contour-aware enhancement after backbone stage outputs.

Within the residual blocks, the InceptionDWConv2d module from InceptionNeXt is adapted to the residual branch of PResNet and denoted as DIDM (Yu et al., 2024). DIDM divides the input feature X along the channel dimension into a preserved branch, a local square branch, a horizontal strip branch, and a vertical strip branch:


$$\left[X_{\text{id}},X_{\text{s}},X_{\text{w}},X_{\text{h}}\right]=\text{Split}(X).$$

where *X*_id_ denotes the preserved branch, which is used to maintain the original feature distribution; *X*_s_, *X*_w_, and *X*_h_ represent the local square branch, the horizontal strip branch, and the vertical strip branch, respectively. Then, the three spatial mixing branches employ 3×3, 1×k, and k×1 depthwise convolutions to extract texture information in different orientations:


$$X_{\text{dw}}=\mathrm{Concat}\left(X_{\text{id}},\text{DW}{\mathrm{Conv}}_{3\times 3}\left(X_{\text{s}}\right),\text{DW}{\mathrm{Conv}}_{1\times k}\left(X_{\text{w}}\right),\text{DW}{\mathrm{Conv}}_{k\times 1}\left(X_{\text{h}}\right)\right),$$

The concatenated feature is then processed by a 1×1 convolution, batch normalization, and an activation function for channel reorganization:


$${\text{Y}}_{\text{didm}}=\delta \left(\text{BN}\left({\mathrm{Conv}}_{1\times 1}\left(X_{\text{dw}}\right)\right)\right),$$

where δ(·) denotes the activation function, and *Y*_didm_ represents the output feature of DIDM. Through this design, DIDM preserves part of the original channel information while introducing local, horizontal, and vertical spatial responses through depthwise convolutions. In this study, its main role is to provide a low-cost directional texture supplement within the PResNet residual branch, rather than increasing model complexity through heavy spatial modeling. This design is particularly useful for representing rice lesions that appear as local spots or stripe-like symptoms along the leaf direction.

After DIDM performs lightweight direction-aware spatial mixing within the residual blocks, the LCGA-Backbone further embeds CEGA Blocks after the C3, C4, and C5 stage outputs. Here, C3, C4, and C5 denote the feature maps generated by the third, fourth, and fifth stages of the PResNet backbone, respectively. As the network depth increases, their spatial resolutions progressively decrease, while their receptive fields and levels of semantic abstraction gradually increase. C3 retains relatively rich edge, texture, and localization information, making it important for preserving small lesions and weak boundaries. C4 provides an intermediate representation that balances spatial details with contextual semantics, and is therefore suitable for describing irregular lesion morphology and local structural relationships. C5 contains stronger category-level semantics and broader contextual information, which contribute to disease-class discrimination and background-interference suppression. Applying CEGA after all three stage outputs therefore enables complementary enhancement of fine contours, lesion structures, and high-level semantics. This strategy preserves the original feature scales and channel interfaces while providing more discriminative multi-level features for subsequent fusion in the MEIF-Neck.

**Figure 2:**
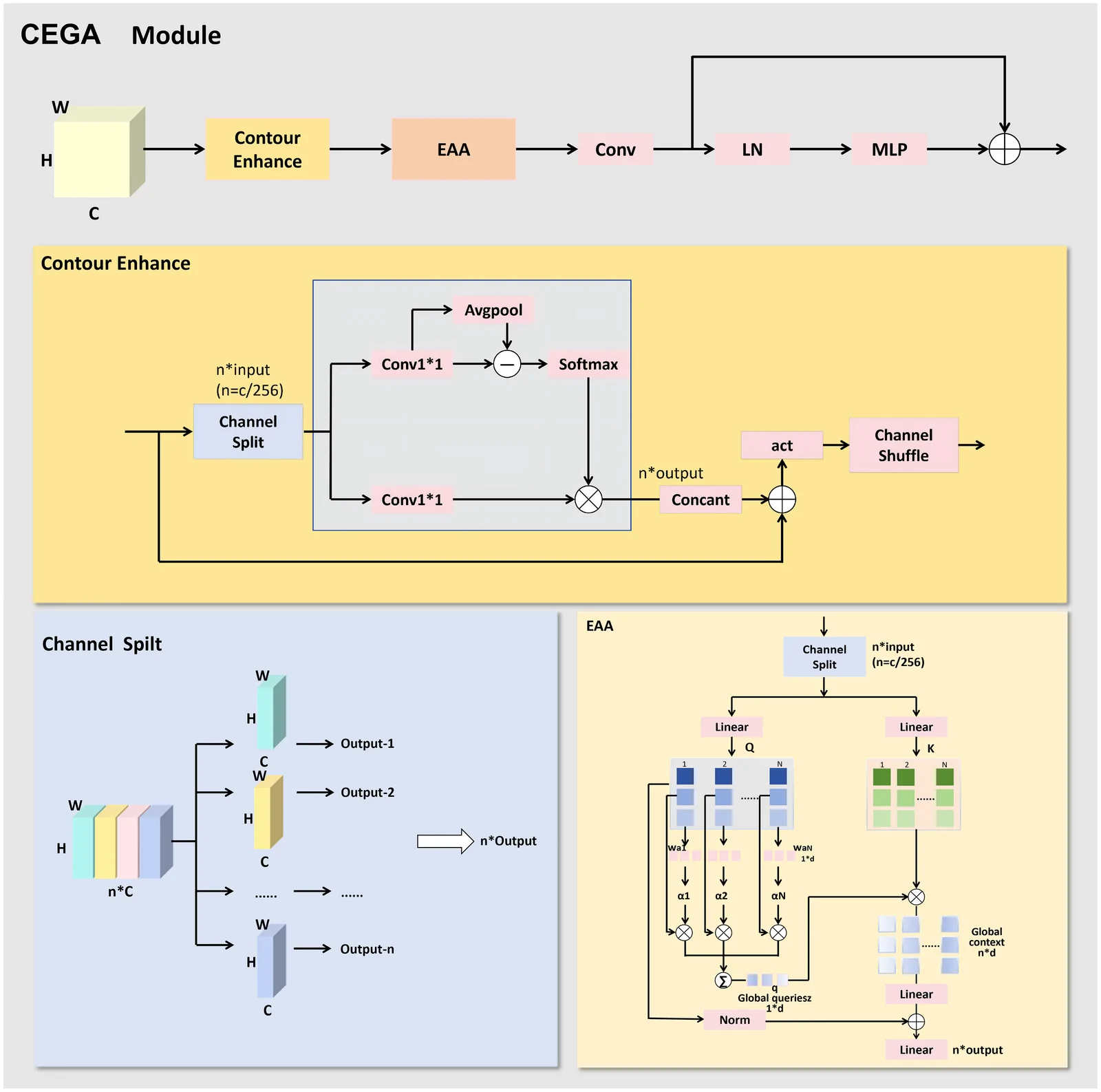
Structure of the CEGA Block.


As shown in **Figure 2**, the CEGA Block extends contour-aware feature enhancement by combining the Contour Enhance Module (CEM), Channel Shuffle, Grouped Efficient Additive Attention (Group-EAA), and Bottleneck Convolutional MLP (Bottleneck ConvMLP). Let the input feature be denoted as X. CEM first obtains the projected feature *X*_p_, then applies local average pooling to extract the low-frequency feature *X*_1_, and obtains the high-frequency contour response *X*_e_ through a difference operation. This process can be simplified as follows:

$$X_{\text{p}}=\phi_{\text{p}}(X),$$

$$X_{\text{l}}=\mathrm{AvgPool}\left(X_{\text{p}}\right),$$

$$X_{\text{e}}=X_{\text{p}}-X_{\text{l}},$$

$$X_{\text{c}}=\sigma \left(X+\phi_{\text{b}}(X)⊙X_{\text{e}}\right).$$

In this process, *ϕ*_p_ and *ϕ*_b_ denote the intra-group projection and contour modulation mapping, respectively, ⊙ denotes element-wise multiplication, and σ represents the activation function. Compared with direct full-channel mapping, CEM adopts dynamic grouped convolution for contour enhancement, which preserves intra-group channel interaction while reducing redundant computation. The contour response *X*_e_, obtained by local smoothing-based differencing, emphasizes lesion boundaries, spot contours, and texture transition regions, thereby improving the representation of weak-boundary lesions in deep features.

After obtaining the contour-enhanced feature, the CEGA Block further applies Channel Shuffle to alleviate inter-group isolation caused by grouped processing. Group-EAA is then used to model long-range contextual dependencies within each group. Finally, Bottleneck ConvMLP performs lightweight nonlinear channel mixing after normalization:


$$X_{\text{s}}=\mathrm{Shuffle}\left(X_{\text{c}}\right),$$

$$X_{\text{g}}=\mathrm{GroupEAA}\left(X_{\text{s}}\right),$$

$$Y=X_{\text{g}}+\text{MLP}\left(\text{LN}\left(X_{\text{g}}\right)\right).$$

Here, *X*_s_ denotes the feature after channel shuffle, *X*_g_ represents the contextual feature aggregated by Group-EAA, LN(·) denotes LayerNorm2d, MLP(·) denotes the Bottleneck ConvMLP, and *Y* is the final output of the CEGA Block. In summary, DIDM focuses on supplementing direction-sensitive local spatial mixing within residual blocks, whereas the CEGA Block performs contour enhancement, channel shuffle, grouped contextual aggregation, and nonlinear channel mixing after stage outputs. By combining DIDM with the contour-enhanced EAA mechanism in CEGA, LCGA-Backbone improves the representation of directional lesion textures, weak boundaries, and contextual semantics while keeping the additional computational overhead controllable.

### 3.2 MEIF-Neck

The HybridEncoder in the original RT-DETRv2 performs multi-scale feature fusion through a top-down FPN and a bottom-up PAN. In this process, high-level semantic features are first processed by lateral convolution and upsampling, and then concatenated with low-level texture features. The concatenated features are subsequently fed into a fusion module for channel compression and local reconstruction. Although this design is simple and efficient, it lacks sufficient interaction modeling for the complete 2C-channel concatenated features and does not explicitly select salient lesion regions. For small scattered lesions such as brown spot, shallow details are easily weakened during feature fusion. For weak-boundary or stripe-like lesions such as bacterial leaf blight and rice blast, simple concatenation and reconstruction are also susceptible to interference from leaf veins, shadows, and complex background textures.

**Figure 3:**
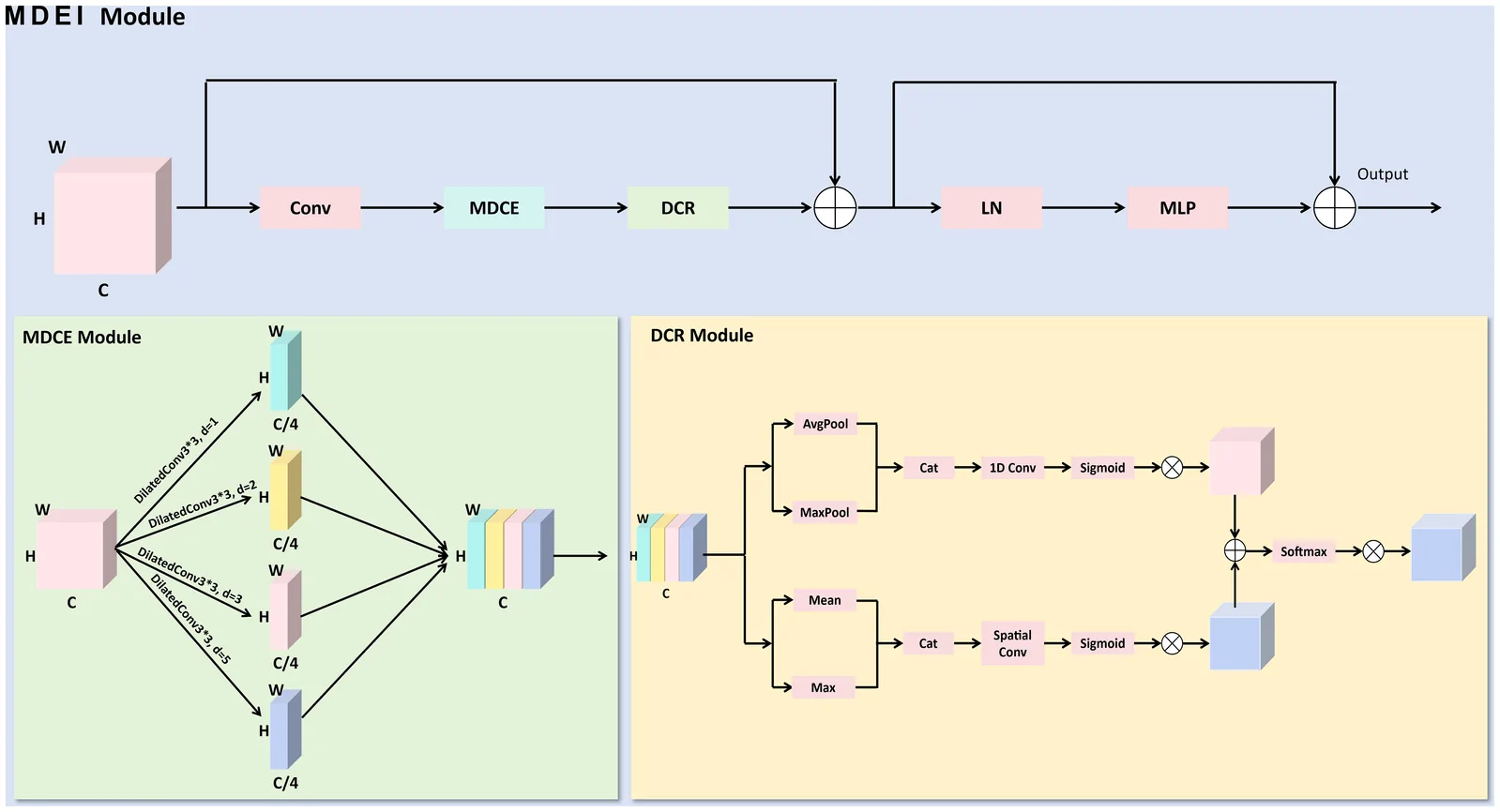
Structure of the MDEI module.

**Figure 4:**
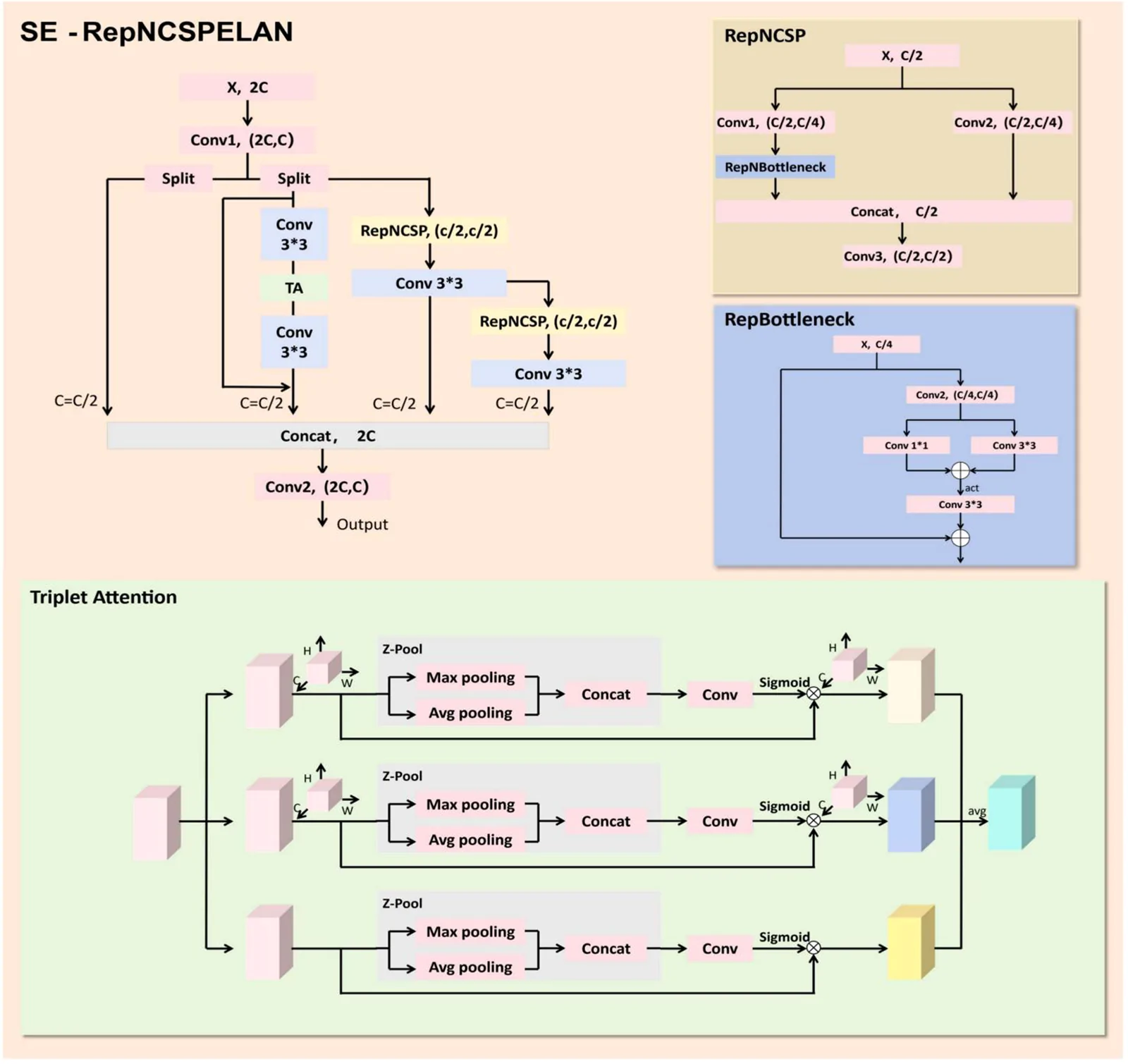
Structure of the SE-RepNCSPELAN module.

To address these problems, this study constructs the MEIF-Neck. The core idea is to reorganize the fusion process according to an “evidence interaction first, lightweight reconstruction later” strategy in the top-down FPN pathway, so that high-level semantic information and low-level lesion textures can interact more fully before channel compression. Specifically, after the high-level feature is upsampled, it is concatenated with the lateral feature at the current scale to form a 2C-channel fused feature. This feature is first fed into MDEI for multi-scale contextual extraction and salient evidence competition, and is then processed by SE-RepNCSPELAN for lightweight fusion reconstruction. The structures of MDEI and SE-RepNCSPELAN are shown in **Figure 3** and **Figure 4**, respectively.

Let *P*_i_ denote the lateral feature at the current scale, *F*_i+1_ denote the output feature from the preceding higher-level stage, and *ψ*_i_(·) denote the 1×1 lateral convolution. The top-down fusion process of FPN can be formulated as follows:


$${\tilde{F}}_{i+1}=\psi_{i}\left(F_{i+1}\right),$$

$$Z_{i}=Concat\left(UP\left({\tilde{F}}_{i+1}\right),P_{i}\right),$$

$${\hat{Z}}_{i}=MDEI\left(Z_{i}\right),$$

$F_{i}=\text{SE−RepNCSPELAN}\left({\hat{Z}}_{i}\right).$Here, ${\tilde{F}}_{i+1}$ represents the high-level feature adjusted by lateral convolution, Up(·) denotes the upsampling operation, Concat(·) denotes channel-wise concatenation, *Z*_i_ represents the 2C-channel fused feature after cross-scale concatenation, ${\hat{Z}}_{i}$ denotes the fused feature enhanced by MDEI, and *F*_i_ is the top-down output feature at the current scale. Since MDEI is placed before channel compression, it can directly process the complete 2C-channel fused feature, thereby preventing inadequate interaction between high-level semantics and low-level textures caused by premature channel compression.

MDEI is introduced to enrich multi-scale contextual information and salient lesion evidence before fusion reconstruction, especially for small scattered lesions and weak-boundary regions. Given the input feature *Z*_i_, the module first applies a 1×1 convolution for preprocessing to obtain the basic fused feature *U*_i_. Then, multi-scale dilated contextual extraction is performed to generate contextual responses *M*_i_ under different receptive fields. Next, a dynamic competitive recalibration mechanism is used to select salient responses from the multi-scale responses, resulting in the recalibrated feature *R*_i_. Finally, LN and MLP are employed for lightweight channel mixing. This process can be expressed as follows:


$$U_{\text{i}}=\psi_{1\times 1}\left(Z_{\text{i}}\right),$$

$$M_{\text{i}}=\text{MDCE}\left(U_{\text{i}}\right),$$

$$R_{\text{i}}=U_{\text{i}}+\text{DCR}\left(M_{\text{i}}\right),$$

$${\hat{Z}}_{\text{i}}=R_{\text{i}}+\text{MLP}\left(\text{LN}\left(R_{\text{i}}\right)\right).$$

In the above equations, MDCE(·) is used to extract multi-scale dilated contextual information, while DCR(·) reallocates response weights according to channel evidence and spatial evidence. Branches with smaller dilation rates focus more on lesion boundaries and local textures, whereas branches with larger dilation rates are used to perceive surrounding lesion regions, leaf-vein backgrounds, and the relationships among adjacent lesions. Through this “multi-scale extraction–evidence competition–residual mixing” strategy, MDEI highlights discriminative lesion regions at the early stage of fusion, which is beneficial for small brown spot lesions, weak rice blast boundaries, and lesion regions disturbed by leaf-vein backgrounds.

Building on a YOLO-style feature fusion structure, SE-RepNCSPELAN is used for lightweight reconstruction of the 2C-channel fused feature enhanced by MDEI. Let its input be denoted as ${\hat{Z}}_{\text{i}}$. The module first uses a 1×1 convolution to compress the number of channels from 2C to C, and then splits the compressed feature into a preserved branch $T_{i}^{k}$ and a trainable branch $T_{i}^{t}$ along the channel dimension:


$$T_{i}=\rho_{1\times 1}\left({\hat{Z}}_{i}\right)$$

$$\left[T_{i}^{k},T_{i}^{t}\right]=Split\left(T_{i}\right),$$

The preserved branch directly transmits basic texture information to reduce excessive smoothing of small lesion details caused by successive convolutions. The trainable branch is simultaneously fed into the progressive reconstruction path and the spatial evidence residual path. The progressive reconstruction path gradually extracts local contextual information and structured semantic features through two cascaded RepNCSP3x3Stem modules:


$$T_{i}^{1}=RepStem_{1}\left(T_{i}^{t}\right),$$

$$T_{i}^{2}=RepStem_{2}\left(T_{i}^{1}\right).$$

The spatial evidence residual path models channel–spatial interactions through 3×3 convolution, Triplet Attention, and another 3×3 convolution, and forms a residual enhancement with the trainable branch:


$$T_{i}^{s}=T_{i}^{t}+\gamma_{2}\left(\text{TA}\left(\gamma_{1}\left(T_{i}^{t}\right)\right)\right),$$

where γ_1_(·) and γ_2_(·) denote the two 3×3 convolution mappings before and after Triplet Attention, respectively. TA(·) represents Triplet Attention, and $T_{i}^{s}$ denotes the spatial-evidence-enhanced feature of the trainable branch. Finally, the preserved branch, the spatial-evidence-enhanced branch, and the two progressive reconstruction branches are concatenated and compressed back to C channels through a 1×1 convolution:


$$F_{i}=\eta_{1\times 1}\left(Concat\left(T_{i}^{k},T_{i}^{s},T_{i}^{1},T_{i}^{2}\right)\right),$$

Through the above design, SE-RepNCSPELAN preserves basic texture information while introducing spatial evidence compensation and progressive local reconstruction. This helps reduce the influence of leaf veins and background textures during feature fusion and improves the representation of small-scale and weak-boundary lesions. In the bottom-up PAN pathway, MDEI is not repeatedly introduced. Instead, ADown is adopted for downsampling. The downsampled feature is concatenated with the FPN output feature at the corresponding scale and then fed into SE-RepNCSPELAN for reconstruction:


$$D_{i}=ADown\left(O_{i}\right),$$

$$Z_{i+1}^{p}=Concat\left(D_{i},F_{i+1}\right),$$

$$O_{i+1}=\text{SE−RepNCSPELAN}\left(Z_{i+1}^{p}\right),$$

where *O*_i_ denotes the input feature at the current scale in the PAN pathway, *D*_i_ represents the downsampled feature generated by ADown, $Z_{i+1}^{p}$ denotes the bottom-up concatenated feature, and O_i+1_ represents the output feature of the PAN pathway. This design enables the FPN pathway to focus on evidence interaction and salient-region selection over the complete 2C-channel fused feature, while the PAN pathway focuses on cross-level information transmission and lightweight reconstruction. The two pathways are therefore functionally complementary.

In summary, MEIF-Neck establishes an “evidence interaction–spatial enhancement–progressive reconstruction” fusion process through MDEI and SE-RepNCSPELAN. MDEI directly processes the complete cross-scale feature after FPN concatenation, strengthening the interaction between high-level semantics and low-level textures. SE-RepNCSPELAN serves as a lightweight fusion reconstruction module in both the FPN and PAN pathways, further supplementing spatial evidence and local context. Compared with the direct concatenation and convolutional reconstruction strategy in the original HybridEncoder, MEIF-Neck can more fully exploit shallow texture details and high-level semantic information, thereby improving the representation quality of rice lesion features under small-scale, weak-boundary, and complex-background conditions.

### 3.3 DAD-DA

The decoder of the original RT-DETRv2 adopts multi-scale deformable attention to aggregate image features around reference points. In this process, each object query directly predicts two-dimensional sampling offsets. Although this strategy is efficient, the sampling direction and sampling amplitude are coupled within the same offset vector. When rice lesions show slender shapes, irregular contours, or weak boundaries, such coupled offsets may be sensitive to the aspect ratio of the reference box, resulting in unstable sampling locations. To address this problem, this study introduces Direction-Amplitude Decoupled Deformable Attention (DAD-DA). Instead of directly predicting a two-dimensional offset, DAD-DA adjusts the sampling formulation by decomposing the sampling offset into a direction component and a radius component:


$$\Delta p_{i,h,l,k}=r_{i,h,l,k}u_{i,h,l,k}$$

$$p_{i,h,l,k}=c_{i,l}^{ref}+\Delta p_{i,h,l,k}$$

where *q_i_* denotes the *i*-th object query, *h* denotes the attention head index, *l* denotes the feature level index, *k* denotes the sampling point index, $u_{i,h,l,k}$ is the sampling direction, $r_{i,h,l,k}$ is the sampling radius, and $c_{i,l}^{ref}$ is the center of the reference box.

First, a basic direction template is constructed for each attention head:


$$\theta_{h}=\frac{2\pi \left(h-1\right)}{H}$$

$$b_{h}=\frac{\left(\cos\theta_{h},\sin\theta_{h}\right)}{\max\left(\left|\cos\theta_{h}\right|,\left|\sin\theta_{h}\right|\right)+\epsilon }$$

where *H* is the number of attention heads, and ϵ is a small constant. The L∞-normalized direction template preserves the square-like initial sampling distribution of deformable attention. Then, the query predicts a rotation residual parameter:


$$\beta_{i,h,l,k}=W_{rot}q_{i}+b_{rot}$$

The corresponding normalized rotation terms are defined as:


$$c_{i,h,l,k}=\frac{1}{\sqrt{1+\beta_{i,h,l,k}^{2}}+\epsilon }$$

$$s_{i,h,l,k}=\frac{\beta_{i,h,l,k}}{\sqrt{1+\beta_{i,h,l,k}^{2}}+\epsilon }$$

Thus, the rotation matrix is formulated as:


$$R\left(\beta_{i,h,l,k}\right)=\left[\begin{array}{cc} c_{i,h,l,k} & -s_{i,h,l,k}\\ s_{i,h,l,k} & c_{i,h,l,k} \end{array}\right]$$

For a reference box (*c_x_,c_y_,w,h*), its horizontal and vertical semi-axes are defined as:


$$a_{x}=\frac{w}{2},$$

$$a_{y}=\frac{h}{2}$$

To reduce the direct influence of the reference-box aspect ratio on direction learning, DAD-DA introduces a geometric compression-restoration strategy. The aspect ratio and compression coefficients are defined as:


$$\rho =\frac{\max\left(a_{x},a_{y}\right)}{\min\left(a_{x},a_{y}\right)+\epsilon }$$

$$\kappa_{x}=\{\begin{array}{cc} \rho , & a_{x}\geq a_{y}\\ 1, & a_{x}<a_{y} \end{array},\;\kappa_{y}=\{\begin{array}{cc} 1, & a_{x}\geq a_{y}\\ \rho , & a_{x}<a_{y} \end{array}$$

The basic direction is first mapped into the compressed reference-box space:


$${\tilde{b}}_{h}=\left(\frac{b_{h}^{x}}{\kappa_{x}+\epsilon },\frac{b_{h}^{y}}{\kappa_{y}+\epsilon }\right)$$

Then, direction rotation is performed in the compressed space, and the learned direction residual is restored to the original reference-box space:


$${\tilde{d}}_{i,h,l,k}=R\left(\beta_{i,h,l,k}\right){\tilde{b}}_{h}$$

$$\Delta d_{i,h,l,k}=\left(\kappa_{x}\left({\tilde{d}}_{i,h,l,k}^{x}-{\tilde{b}}_{h}^{x}\right),\kappa_{y}\left({\tilde{d}}_{i,h,l,k}^{y}-{\tilde{b}}_{h}^{y}\right)\right)$$

The restored direction is then normalized to obtain the final sampling direction:


$$u_{i,h,l,k}=\frac{b_{h}+\Delta d_{i,h,l,k}}{\max\left(\left|b_{h}^{x}+\Delta d_{i,h,l,k}^{x}\left|,\right|b_{h}^{y}+\Delta d_{i,h,l,k}^{y}\right|\right)+\epsilon }$$

Through this process, the direction residual is learned in a compressed geometric space and restored according to the reference-box aspect ratio. This reduces the effect of elongated boxes on sampling direction learning without introducing an additional learnable shape restoration branch.

For the sampling radius, DAD-DA calculates the boundary radius along the current sampling direction:


$$r_{i,h,l,k}^{edge}=\min\left(\frac{a_{x}}{\left|u_{i,h,l,k}^{x}\right|+\epsilon },\frac{a_{y}}{\left|u_{i,h,l,k}^{y}\right|+\epsilon }\right)$$

where $r_{i,h,l,k}^{\text{edge}}$ represents the distance from the reference-box center to the box boundary along the current direction. In implementation, this term is detached from the computational graph to provide a stable geometric constraint. Meanwhile, the query predicts a radius gain:


$$\gamma_{i,h,l,k}=Softplus\left(W_{g}q_{i}+b_{g}+b_{0}\right),$$

$$b_{0}=\ln(e-1)$$

where $\gamma_{i,h,l,k}$ denotes the radius gain. The bias *b*_0_ makes the initial value of $\gamma_{i,h,l,k}$ equal to 1 when the gain branch is zero-initialized. Given *K_l_* sampling points at the *l*-th feature level, the final sampling radius is defined as:


$$r_{i,h,l,k}=k\cdot \frac{1}{K_{l}}\cdot \gamma_{i,h,l,k}\cdot sg\left(r_{i,h,l,k}^{edge}\right)$$

where *sg*(·) denotes the stop-gradient operation. Therefore, the final sampling location is determined by the decoupled direction $u_{i,h,l,k}$ and radius $r_{i,h,l,k}$. Since the boundary radius already contains the scale information of the reference box, no additional (*w,h*) modulation term or global offset scaling coefficient is required.

In implementation, DAD-DA replaces the original sampling offset prediction branch with two lightweight branches, namely the direction rotation residual branch *W_rot_* and the radius gain branch *W_g_*. Both branches are initialized to zero. At the initial training stage, $\beta_{i,h,l,k}=0$ and $\gamma_{i,h,l,k}=1$, which means that the sampling direction follows the basic direction template, while the sampling radius is determined by the basic radius order, the sampling-point scale, and the boundary radius of the reference box. In summary, DAD-DA improves the geometric stability of deformable attention sampling through direction-amplitude decoupling, aspect-ratio-aware geometric compression-restoration, and boundary-radius-constrained sampling, thereby enhancing feature aggregation for slender and irregular rice lesion regions.

## 4 Experiments and analysis

### 4.1 Dataset and experimental settings

Evaluation was conducted under two dataset settings. The constructed rice leaf disease detection dataset served as the primary dataset and contained three disease categories, namely bacterial leaf blight, rice blast, and brown spot. The original images were collected from four publicly available rice disease image resources: Rice leaf disease, Rice_Leaf_AUG, RiceLeafsDisease, and Rice Disease Dataset. These datasets were obtained from public Kaggle dataset pages or their corresponding public data pages. To ensure consistency across different sources, the collected images were reorganized according to their original category names and file naming information, and then manually screened and re-annotated in a unified bounding-box format. Among the four sources, Rice Disease Dataset provides object-detection-style rice leaf disease images, whereas the other three sources mainly provide category-level rice disease images. Therefore, all retained images were processed under the same category definition, annotation rule, image resizing strategy, and evaluation protocol.

To improve the reliability of the constructed dataset, the original images were manually screened before annotation and dataset splitting. Images with obvious stretching, severe distortion, excessive artificial augmentation, abnormal color distribution, severe blurring, or unclear disease symptoms were removed. Considering that public datasets may contain repeated images, augmented samples, or visually similar samples, a manual duplicate-checking procedure was conducted based on source folder names, file names, disease labels, image appearance, leaf posture, lesion distribution, and background similarity. Obvious duplicates, near-duplicate images, and images with highly similar visual content were excluded whenever identified. During sample selection, images with clearer lesion morphology, natural leaf texture, and larger visual differences were preferentially retained to reduce sample redundancy and increase dataset diversity.

After manual screening and reorganization, a total of 750 original images were retained in the constructed dataset, including 228 images of bacterial leaf blight, 236 images of rice blast, and 286 images of brown spot. The source composition of each disease category is shown in **Table 1**.

**Table 1:** Source composition of the constructed rice leaf disease dataset.

Class	Rice leaf disease	Rice_Leaf_AUG	RiceLeafsDisease	Rice disease dataset	Total
Bacterial leaf blight	28	107	76	17	228
Rice blast	33	119	71	13	236
Brown spot	27	145	99	15	286

The constructed dataset was then divided into training, validation, and test sets at a ratio of 7:2:1, and the detailed statistics are shown in **Table 2**.

**Table 2:** Statistics of the rice leaf disease dataset.

Class	Original images	Train	Val	Test	Boxes	Augmented training images
Bacterial leaf blight	228	160	45	23	617	480
Rice blast	236	165	47	24	381	495
Brown spot	286	200	58	28	1252	600
Total	750	525	150	75	2250	1575

After dataset reorganization, all retained images were manually checked and annotated in a unified bounding-box format. The initial disease categories were determined according to the original category names, folder names, and file naming information of the public datasets. Then, under the guidance of teachers familiar with rice disease image recognition, the lesion regions were re-annotated using bounding boxes. During annotation, visible diseased regions were used as the annotation targets, and the bounding boxes were drawn as tightly as possible around the lesion areas. For small and scattered lesions, spatially separated lesion regions were annotated as independent boxes when they could be visually distinguished. When several tiny lesions were densely clustered and difficult to separate reliably, they were annotated as one local lesion region. For slender or stripe-like lesions, the bounding box was drawn along the main visible lesion region while minimizing the inclusion of healthy leaf tissue. For multiple lesions on the same leaf, each visually separable lesion region was annotated separately. Images with highly ambiguous symptoms, inconsistent source labels, severe occlusion, or possible mixed infection were excluded unless the dominant symptom could be clearly assigned to one of the three target categories after manual checking.

**Figure 5:**
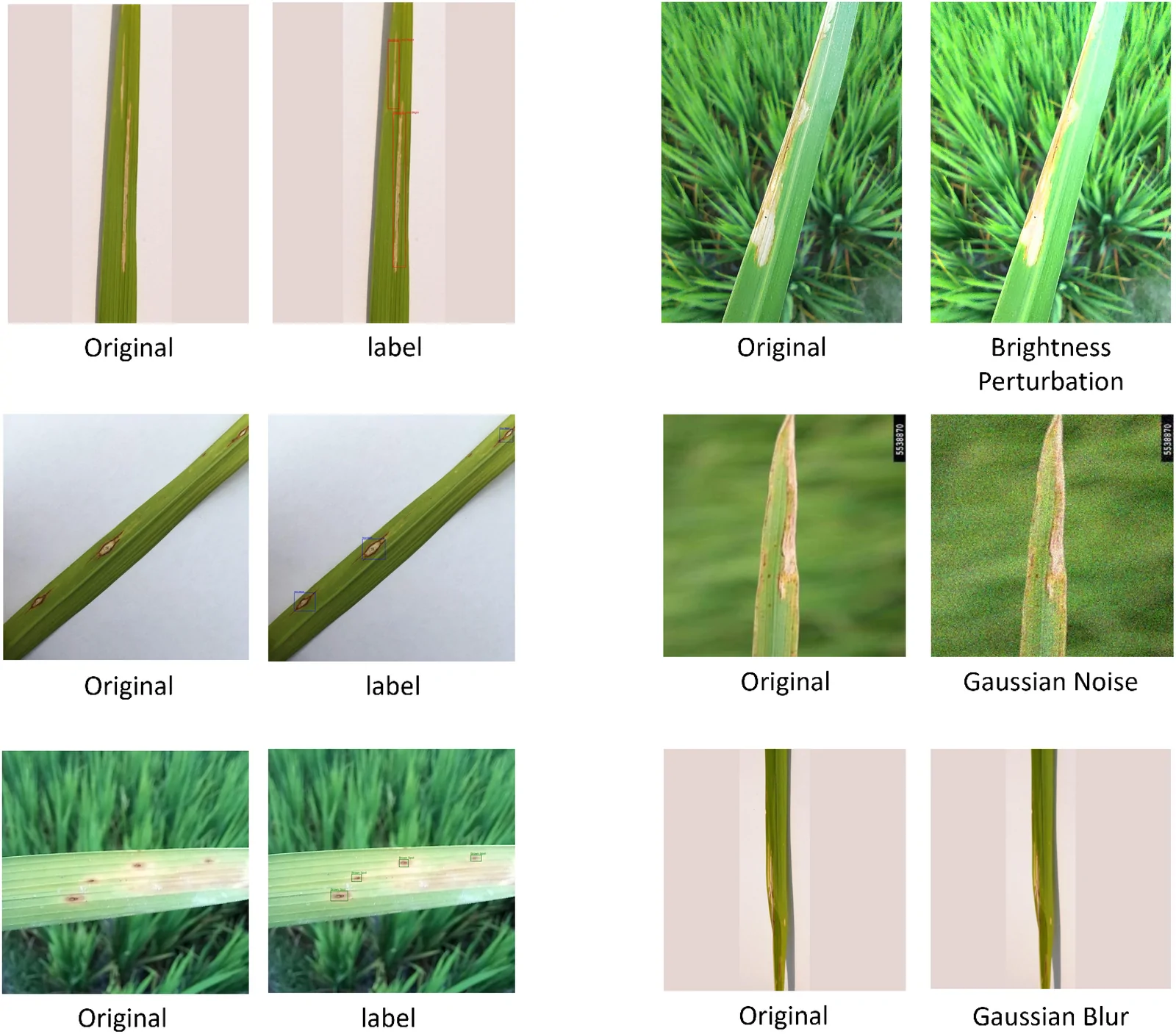
Examples of annotation and data augmentation.

To improve the adaptability of the model to different imaging conditions while avoiding geometric distortions that could alter lesion morphology and leaf texture, brightness adjustment, noise perturbation, and slight blurring were adopted for training data augmentation. During augmentation, the original training images were retained, and two augmented images were generated for each training image. Finally, the training set was expanded to three times its original size. The image augmentation results are shown in **Figure 5**.

The second dataset setting was the public Rice Disease Dataset, which was used as an additional benchmark dataset in the comparative experiments. This dataset contains the same three disease categories as the constructed dataset, including bacterial leaf blight, rice blast, and brown spot, and provides object-detection-style annotations. To ensure consistency between the two dataset settings, all compared models were trained and evaluated using the same input size and evaluation metrics. The results on the public Rice Disease Dataset are reported separately in **Table 4**, while the main comparison results on the constructed dataset are reported in **Table 5**.

**Table 3:** Experimental parameter configuration.

Configuration item	Parameter
Operating system	Windows 10, version 10.0.26100
Python	3.9.23
Deep learning framework	PyTorch 2.2.2
GPU	NVIDIA GeForce RTX 4060 Laptop GPU
GPU memory	8 GB
Number of GPUs	1
NVIDIA Driver	577.00
CUDA Driver Version	12.9
PyTorch CUDA Version	11.8
cuDNN	8700
Input size	640×640

**Table 4:** Additional experimental results on the public Rice Disease Dataset.

Model	BLB	Blast	BS	AP	AP50	AP75	Params/M	GFLOPs
RT-DETRv2-R50	71.4%	73.6%	69.5%	29.8%	71.5%	19.8%	42.7	67.9
D-FINE-m	70.5%	72.0%	62.4%	28.8%	68.3%	16.8%	19.2	56.3
YOLOv10-m	79.4%	71.5%	62.4%	28.4%	71.1%	17.6%	15.4	59.1
YOLO11-m	80.2%	76.0%	65.2%	30.2%	73.8%	19.6%	20.1	68
YOLO12-m	80.9%	74.1%	68.5%	30.8%	74.5%	20.5%	20.2	67.5
YOLO26-m	81.2%	75.1%	69.0%	31.2%	75.1%	20.9%	20.4	68.2
CED-RTDETR	78.6%	74.8%	73.3%	34.1%	77.1%	23.2%	45.6	71.1

**Table 5:** Main comparison results on the constructed rice leaf disease dataset.

Model	BLB	Blast	BS	AP	AP50	AP75	Params/M	GFLOPs
RT-DETRv2-R50	73.0%	68.9%	69.0%	25.4%	70.3%	13.8%	42.7	67.9
D-FINE-m	69.6%	72.9%	70.8%	25.8%	71.1%	15.1%	19	56.3
YOLOv10-m	63.9%	79.1%	65.2%	23.9%	69.4%	12.4%	15.4	59.1
YOLO11-m	68.4%	76.9%	70.4%	24.8%	71.9%	14.7%	20.1	68
YOLO12-m	70.5%	73.7%	73.0%	25.6%	72.4%	14.9%	20.2	67.5
YOLO26-m	72.9%	72.7%	73.7%	25.9%	73.1%	15.6%	20.4	68.2
CED-RTDETR	75.4%	73.6%	76.7%	29.5%	75.2%	17.6%	45.6	71.1

The experimental environment and parameter configuration are summarized in **Table 3**. The input image size was uniformly set to 640 × 640. The evaluation metrics included AP, AP50, AP75, Params, and GFLOPs. AP denotes the average precision calculated over IoU thresholds ranging from 0.50 to 0.95 with an interval of 0.05. AP50 and AP75 represent the average precision at IoU thresholds of 0.50 and 0.75, respectively. Params and GFLOPs are used to measure model complexity. In addition, PR curves with recall as the horizontal axis were plotted to analyze the model’s ability to maintain precision at different recall levels and its detection stability for lesion targets.

### 4.2 Comparative experiments

To evaluate the proposed method more comprehensively, experiments were conducted under two dataset settings. The first setting used the constructed rice leaf disease dataset, which was divided into training, validation, and test sets at a ratio of 7:2:1. The second setting used the public Rice Disease Dataset as an additional benchmark dataset. These two settings were used to compare different detection models under unified image resizing, training, and evaluation protocols. Therefore, the experimental analysis in this section focuses on dataset-level detection performance, while broader cross-source and field-level validation will be further investigated in future work.

To verify the effectiveness of the proposed method, YOLOv10-m (Wang et al., 2024), YOLO11-m (Jocher and Qiu, 2024), YOLO26-m (Jocher et al., 2026), RT-DETRv2-R50 (Lv et al., 2024), D-FINE-m (Peng et al., 2024), and YOLO12-m (Tian et al., 2025) were selected as comparative models. All models were trained and evaluated using the same input size within each dataset setting. The additional experimental results on the public Rice Disease Dataset are shown in **Table 4**, and the main comparison results on the constructed rice leaf disease dataset are shown in **Table 5**.

As shown in **Table 4**, CED-RTDETR achieves the best overall performance on the public Rice Disease Dataset, with AP, AP50, and AP75 values of 34.1%, 77.1%, and 23.2%, respectively. Compared with RT-DETRv2-R50, the proposed method improves AP, AP50, and AP75 by 4.3, 5.6, and 3.4 percentage points, respectively. Compared with YOLO26-m, which obtains the strongest overall performance among the non-proposed models, CED-RTDETR still improves AP, AP50, and AP75 by 2.9, 2.0, and 2.3 percentage points, respectively. In terms of class-wise AP50, CED-RTDETR achieves the best result for brown spot, while maintaining competitive performance for bacterial leaf blight and rice blast. These results indicate that the proposed model improves overall detection accuracy and localization quality on the public dataset, rather than relying only on gains in a single disease category.

As shown in **Table 5**, the proposed method achieves the best overall results on the constructed rice leaf disease dataset in terms of AP, AP50, and AP75. Compared with the RT-DETRv2-R50 baseline, CED-RTDETR improves AP from 25.4% to 29.5%, AP50 from 70.3% to 75.2%, and AP75 from 13.8% to 17.6%, corresponding to increases of 4.1, 4.9, and 3.8 percentage points, respectively. These results indicate that the proposed method improves both low-threshold lesion detection performance and higher-threshold localization quality under the current dataset setting.

In terms of class-wise AP50 on the constructed dataset, CED-RTDETR achieves 75.4% for bacterial leaf blight and 76.7% for brown spot, both of which are the highest values among all compared models. Compared with RT-DETRv2-R50, the AP50 values for these two categories increase by 2.4 and 7.7 percentage points, respectively. The improvement for brown spot is particularly pronounced and is consistent with the small, scattered, and weak-boundary characteristics of this disease category. This suggests that the contour-aware backbone and multi-scale evidence fusion neck can enhance the representation of small lesion textures and boundary-related features. For rice blast, CED-RTDETR achieves an AP50 of 73.6%, which is 4.7 percentage points higher than RT-DETRv2-R50, although it is lower than several YOLO-based models in this single category. However, CED-RTDETR obtains the best AP, AP50, and AP75 values overall, indicating that the proposed method provides a more balanced gains in overall detection accuracy and localization quality across the three disease categories. Compared with D-FINE-m and the YOLO-series models, CED-RTDETR maintains the highest overall AP, AP50, and AP75 on the constructed dataset. Among the non-proposed models, YOLO26-m achieves the closest overall AP, AP50, and AP75 values of 25.9%, 73.1%, and 15.6%, respectively. Compared with YOLO26-m, CED-RTDETR improves AP, AP50, and AP75 by 3.6, 2.1, and 2.0 percentage points, respectively. Compared with RT-DETRv2-R50, the proposed method incurs a moderate increase in model complexity, with Params increasing from 42.7M to 45.6M and GFLOPs increasing from 67.9 to 71.1. This indicates that the proposed structural enhancements improve detection accuracy and localization quality while keeping the computational cost within a controllable range.

### 4.3 Ablation experiments

To verify the effectiveness of each proposed module, RT-DETRv2-R50 was used as the baseline, and LCGA-Backbone, MEIF-Neck, and DAD-DA were progressively introduced. The ablation results of the three main components are shown in **Table 6**, and the finer-grained ablation results of CEGA, DIDM, MDEI, and SE-RepNCSPELAN are shown in **Table 7**.

**Table 6:** Ablation experimental results.

Series	LCGA-Backbone	MEIF-Neck	DAD-DA	AP	AP50	AP75	Params/M	GFLOPs
0	×	×	×	25.4%	70.3%	13.8%	42.7	67.9
1	✓	×	×	26.5%	71.6%	14.4%	48.2	72.1
2	✓	✓	×	27.9%	73.4%	16.3%	45.6	71.1
3	✓	✓	✓	29.5%	75.2%	17.6%	45.6	71.1

**Table 7:** Finer-grained ablation experimental results.

Series	CEGA	DIDM	MDEI	SE-RepNCSPELAN	AP	AP50	AP75	Params/M	GFLOPs
0	×	×	×	×	25.4%	70.3%	13.8%	42.7	67.9
1	✓	×	×	×	26.3%	71.2%	14.7%	58.2	85.8
2	✓	✓	×	×	26.5%	71.6%	14.4%	48.2	72.1
3	✓	✓	✓	×	27.3%	72.7%	15.7%	50.6	76.1
4	✓	✓	✓	✓	27.9%	73.4%	16.3%	45.6	71.1

The finer-grained ablation results in **Table 7** further show the contribution of each submodule. Introducing CEGA alone improves AP from 25.4% to 26.3%, indicating that contour-guided enhancement contributes to lesion boundary representation. After DIDM is added, AP and AP50 slightly increase to 26.5% and 71.6%, while Params and GFLOPs decrease from 58.2M and 85.8 to 48.2M and 72.1, respectively, compared with using CEGA alone. This indicates that DIDM mainly contributes to a more efficient spatial modeling process. Although its independent accuracy gain is limited, it supplements local, horizontal, and vertical lesion texture responses with lower computational cost, which is beneficial for the subsequent contour-aware enhancement of LCGA-Backbone. Introducing MDEI further improves AP, AP50, and AP75 to 27.3%, 72.7%, and 15.7%, respectively, showing the benefit of multi-scale evidence interaction before feature reconstruction. Finally, after SE-RepNCSPELAN is added, AP, AP50, and AP75 increase to 27.9%, 73.4%, and 16.3%, respectively, while Params and GFLOPs decrease to 45.6M and 71.1. These results indicate that the proposed submodules are complementary in lesion contour enhancement, directional texture extraction, multi-scale evidence interaction, and lightweight feature reconstruction.

As shown in **Table 6**, each main component contributes to performance gains to varying degrees. Compared with the RT-DETRv2-R50 baseline, introducing LCGA-Backbone alone improves AP, AP50, and AP75 from 25.4%, 70.3%, and 13.8% to 26.5%, 71.6%, and 14.4%, corresponding to increases of 1.1, 1.3, and 0.6 percentage points, respectively. This result indicates that directional texture modeling and contour-context enhancement in the backbone can improve the representation of lesion boundaries, weak textures, and local structures, thereby enabling the model to obtain richer lesion-aware features.

After further introducing MEIF-Neck on the basis of LCGA-Backbone, AP, AP50, and AP75 increase to 27.9%, 73.4%, and 16.3%, respectively, with additional improvements of 1.4, 1.8, and 1.9 percentage points compared with Series 1. The improvement in AP75 is particularly evident, indicating that cross-scale evidence interaction and lightweight fusion reconstruction not only enhance the response of lesion regions but also improve bounding-box localization quality. For rice leaf lesions, high-level semantic information and low-level texture details are highly complementary. MEIF-Neck performs evidence interaction and salient-region competition on the complete 2C-channel concatenated feature before channel compression, which helps reduce interference from leaf-vein textures and complex backgrounds and improves the discriminative ability of fused features. After DAD-DA is finally introduced, AP, AP50, and AP75 reach 29.5%, 75.2%, and 17.6%, respectively. Compared with Series 2, these values are further improved by 1.6, 1.8, and 1.3 percentage points, respectively. Compared with the baseline, the overall improvements are 4.1, 4.9, and 3.8 percentage points. These results indicate that direction–radius decoupled sampling and aspect-ratio-aware geometric compression-restoration further enhance the decoder’s localization and feature-aggregation capabilities, thereby improving the localization stability of slender and irregular-boundary lesion regions. From the perspective of model complexity, the proposed modules introduce only a moderate increase in computational cost. Compared with the RT-DETRv2-R50 baseline, the final model increases Params from 42.7M to 45.6M and GFLOPs from 67.9 to 71.1, while AP, AP50, and AP75 are substantially improved. This indicates that the proposed structures provide clear accuracy gains with a controllable increase in model complexity.

### 4.4 Response visualization analysis

To further investigate the influence of different proposed modules on the model’s attention to lesion regions, representative rice leaf disease images were selected for comparative response visualization. In the heatmaps, red and yellow regions indicate positions with strong model responses, whereas blue regions indicate weak responses. Considering that LCGA-Backbone, MEIF-Neck, and DAD-DA may all affect the response intensity in lesion regions, presenting heatmaps for all three modules would lead to overlapping interpretations of small-lesion enhancement, cross-scale fusion, and slender-lesion localization. Therefore, this section focuses on the individual visualization results of LCGA-Backbone and DAD-DA, and analyzes their effects from the perspectives of small-lesion perception and slender-lesion structural response, respectively.

Specifically, the visualization of LCGA-Backbone focuses on whether the backbone can attend more effectively to small-scale, weak-textured, and scattered lesion regions during feature extraction. In contrast, the visualization of DAD-DA focuses on whether the decoder can form more stable and continuous responses along the extension direction of slender lesions. Since MEIF-Neck mainly acts on cross-scale feature fusion, its contribution is more directly reflected by the improvements in AP and AP50 in the ablation experiments; therefore, its heatmap is not separately displayed to avoid repetitive visualization analysis.

#### 4.4.1 Response visualization of LCGA-Backbone

**Figure 6:**
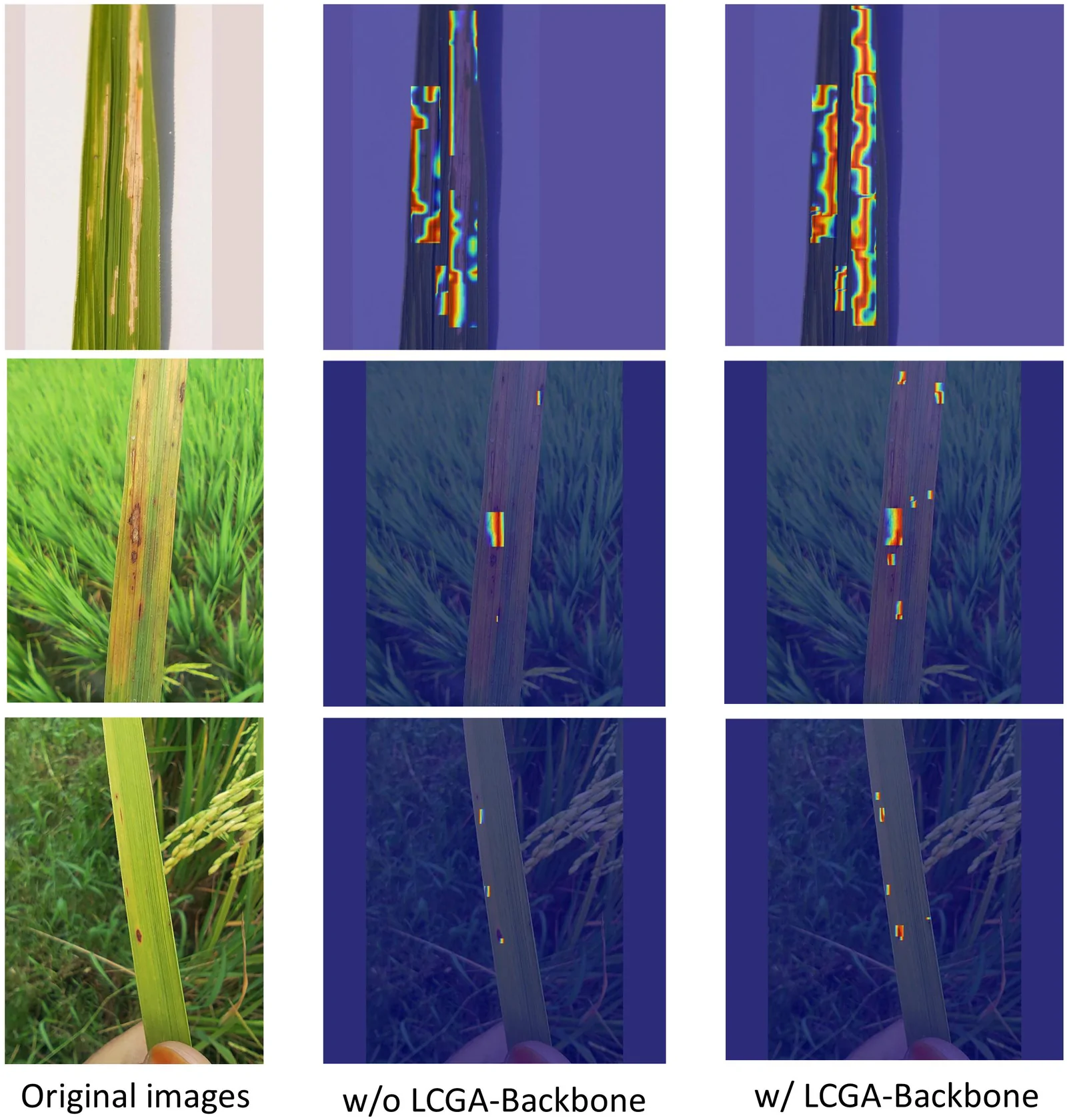
Response visualization comparison of LCGA-Backbone.

**Figure 6** presents the response visualization results before and after introducing LCGA-Backbone. The first column shows the original images, the second column shows the responses of the baseline model without LCGA-Backbone, and the third column shows the responses after introducing LCGA-Backbone. It can be observed that the baseline model generally responds to lesions with relatively large areas or obvious textures. However, for lesions that are sparsely distributed, small in scale, or characterized by weak boundaries, the response is often insufficient. Some small lesions are only sparsely activated or even obscured by leaf texture and background interference.

After introducing LCGA-Backbone, the model shows stronger attention to small lesion regions. For scattered lesions such as brown spot, the improved model produces clearer red and yellow responses at multiple small-scale lesion locations, indicating enhanced sensitivity to local color variations, texture changes, and weak boundary regions. For small but spatially adjacent lesions, the response of the improved model is no longer limited to only a few salient spots; instead, it covers more true lesion regions, demonstrating better perception of small target areas.

This phenomenon is consistent with the structural design of LCGA-Backbone. DIDM introduces lightweight direction-aware spatial mixing within residual blocks, providing richer local texture representation for shallow and middle-level features. CEGA Block further supplements lesion boundary and contextual information after the C3, C4, and C5 stage outputs through contour-difference enhancement, Channel Shuffle, Group-EAA, and Bottleneck ConvMLP. These components jointly enable the backbone to preserve more fine-grained evidence related to small lesions before entering the neck fusion stage. Therefore, the visualization of LCGA-Backbone mainly demonstrates the enhanced attention to small-scale, weak-textured, and scattered lesion regions, rather than emphasizing the continuity of responses along elongated structures.

#### 4.4.2 Response visualization of DAD-DA

**Figure 7:**
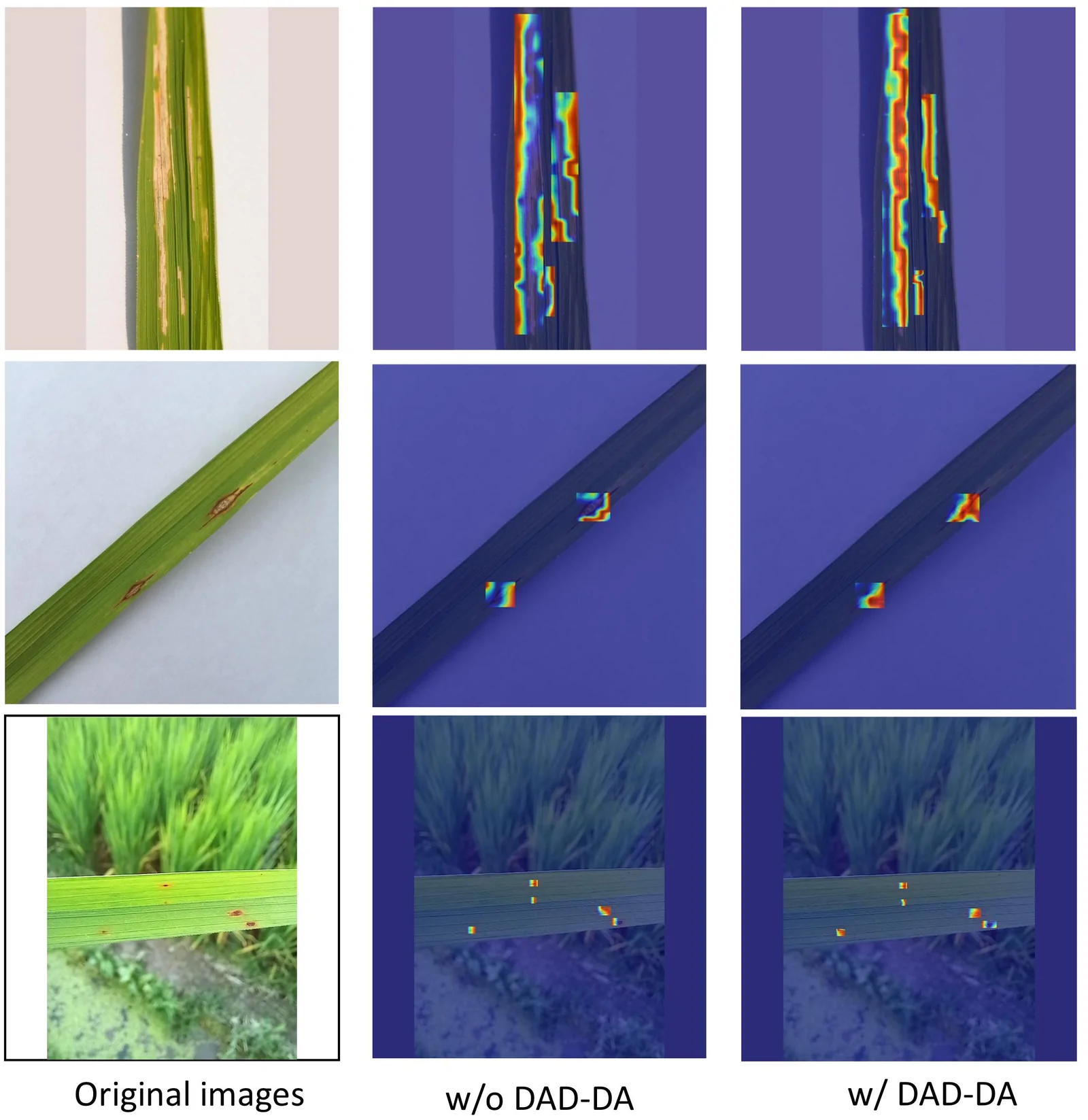
Response visualization comparison of DAD-DA.

**Figure 7** shows the response visualization results before and after introducing DAD-DA. The first column shows the original images, the second column shows the model responses without DAD-DA, and the third column shows the responses after introducing DAD-DA. Unlike LCGA-Backbone, which mainly improves feature perception for small lesion regions, DAD-DA focuses on the geometric stability of deformable attention sampling locations in the decoder. Therefore, its visual differences are mainly reflected in slender lesions such as bacterial leaf blight or lesions extending along leaf veins.

As shown in **Figure 7**, without DAD-DA, the model can still produce high responses on some elongated lesions, but the responses tend to be block-like and discontinuous across local regions. This indicates that the original two-dimensional offset formulation, which learns direction and magnitude simultaneously, is not sufficiently stable in covering the long-axis structure of slender targets. After introducing DAD-DA, the high-response regions are more likely to be continuously distributed along the lesion extension direction, and the heatmap responses are more consistent with the actual morphology of elongated lesions. This is particularly evident in longitudinal chlorotic regions and narrow necrotic regions, where a more complete response chain can be observed.

These results indicate that DAD-DA decomposes the sampling offset into a direction residual and a radius gain, and further introduces aspect-ratio-aware geometric compression-restoration, thereby reducing the direct interference of the reference-box aspect ratio on sampling direction learning. As a result, the sampling points can aggregate effective features more stably along slender lesion regions. Unlike the backbone module, which emphasizes the enhancement of small-lesion feature representation, DAD-DA mainly improves the spatial sampling geometry in the decoding stage, thereby enhancing the structural modeling capability and localization stability for elongated and irregular lesions. In the heatmaps, this is reflected by more continuous long-axis responses, fewer discontinuities, and a response distribution that better matches the actual lesion morphology.

In summary, LCGA-Backbone and DAD-DA have different functional emphases. LCGA-Backbone mainly enhances the attention to small-scale, weak-textured, and scattered lesion regions during the feature extraction stage, enabling the model to capture more fine-grained lesion evidence. DAD-DA mainly improves the sampling stability of the decoder for elongated lesions, allowing the model to form more continuous responses over elongated lesion regions. The two modules complement each other in rice disease detection from the perspectives of feature perception and geometric sampling, respectively, which is consistent with the performance improvements observed in the ablation experiments.

### 4.5 PR curve analysis

**Figure 8:**
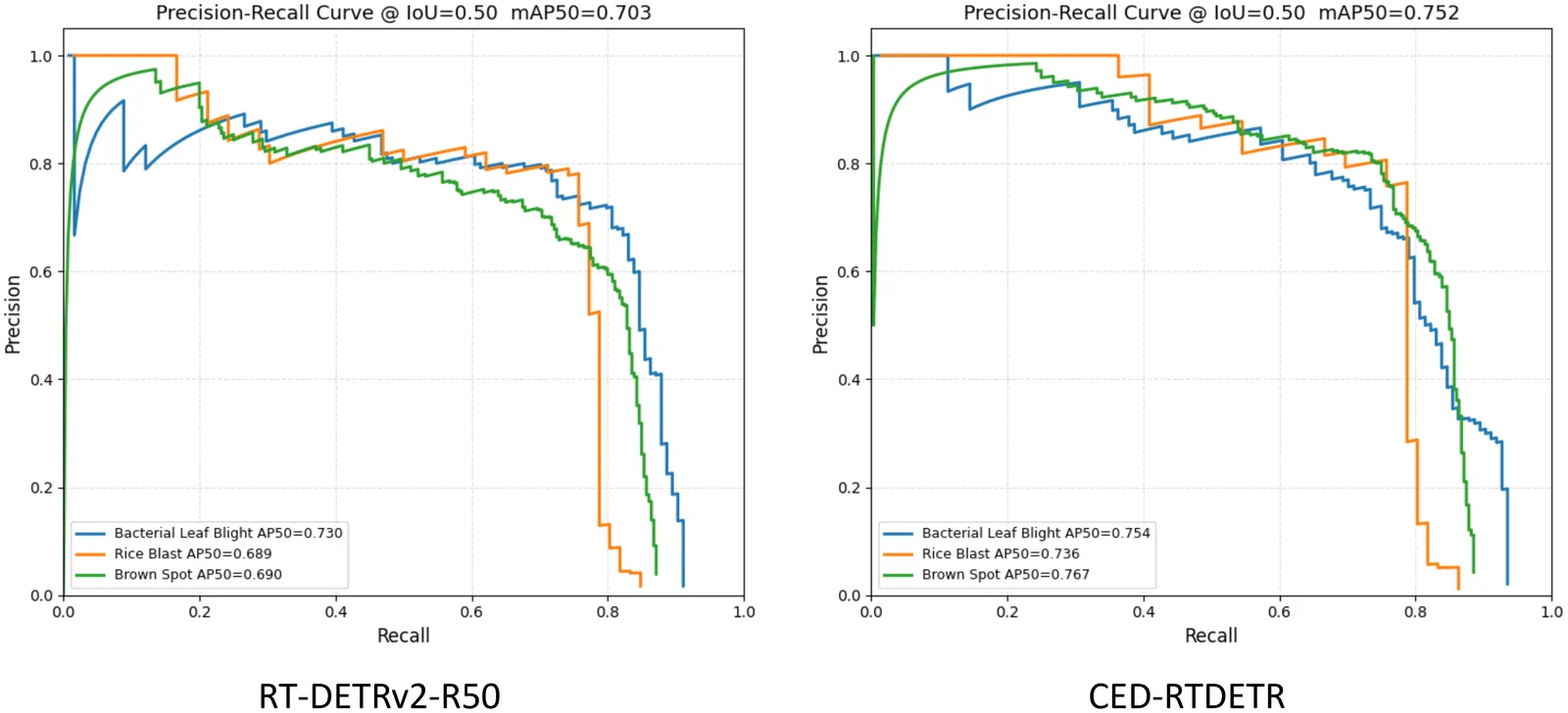
Comparison of PR curves between different models.

To further compare the lesion detection capability of the RT-DETRv2-R50 baseline and the proposed method, the Precision–Recall (PR) curves of both models on the test set of the constructed rice leaf disease dataset were plotted, as shown in **Figure 8**. The PR curve takes recall as the horizontal axis and precision as the vertical axis, reflecting a model’s ability to maintain precision at different recall levels. When the curve maintains high precision over a wide recall range, it indicates that the model can produce more stable responses to real lesion regions. Conversely, a rapid decline in the curve suggests that the model is more likely to introduce false detections or low-quality predicted boxes when recall increases.

As shown in **Figure 8**, the mAP50 of RT-DETRv2-R50 is 0.703, whereas that of the proposed method increases to 0.752. Both the overall area under the curve and the average class-wise performance are improved. From the class-wise curves, the AP50 of bacterial leaf blight increases from 0.730 to 0.754, that of rice blast increases from 0.689 to 0.736, and that of brown spot increases from 0.690 to 0.767. Among them, the improvements for brown spot and rice blast are more pronounced, indicating that the proposed method can effectively improve the detection capability for small scattered lesions and weak-boundary lesions.

In terms of curve shape, the proposed method maintains a higher precision level over a wider recall interval. In particular, as recall gradually increases, the curve of the proposed method exhibits a relatively smoother downward trend, indicating that the model can still maintain reliable predictions while detecting more lesion targets. This phenomenon suggests that the contour and directional texture enhancement provided by LCGA-Backbone, the cross-scale lesion evidence interaction of MEIF-Neck, and the improvement in geometric stability of sampling locations introduced by DAD-DA jointly enhance the response intensity of the model to real lesion regions. Therefore, the proposed method improves recall stability and discriminative responses to lesion regions on the evaluated test images.

### 4.6 Qualitative visualization and failure case analysis

**Figure 9:**
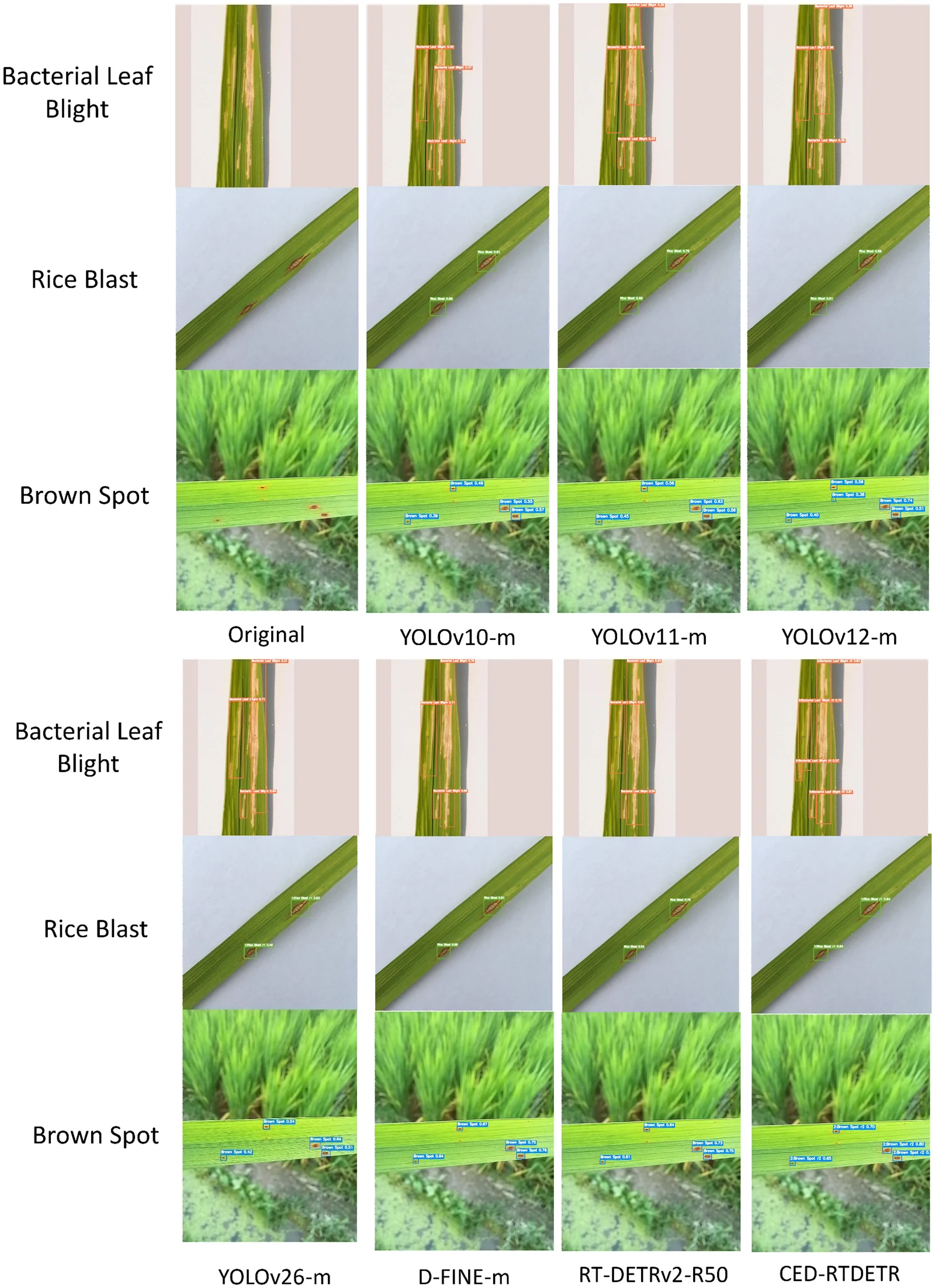
Visual comparison of detection results from different models.

To further analyze the detection performance of different models on various disease categories, the detection results of RT-DETRv2-R50, D-FINE-m, the YOLO series, and CED-RTDETR were visually compared, as shown in **Figure 9**.

From the visualization results, most comparative models can locate the main lesion regions in typical disease images, but clear differences still exist in prediction confidence, small-lesion detection, and bounding-box localization stability. Compared with the baseline model and other comparative models, CED-RTDETR produces more stable detection responses at similar lesion locations. In particular, for small scattered brown spot lesions and weak-boundary rice blast lesions, some comparative models either generate low-confidence predictions or fail to fully cover the lesion regions. In contrast, CED-RTDETR shows clearer correspondence between predicted boxes and visible lesion areas, indicating that the proposed model can more effectively aggregate lesion boundary, texture, and contextual evidence.

This visual difference is consistent with the structural improvements of the proposed method. LCGA-Backbone enhances contour and directional texture representation during backbone feature extraction, MEIF-Neck strengthens cross-scale lesion evidence interaction and suppresses background interference during feature fusion, and DAD-DA improves the geometric stability of deformable attention sampling for slender and irregular lesion regions. Therefore, the detection results in **Figure 9** show that CED-RTDETR can provide more reliable visual localization for small, elongated, and weak-boundary lesions under the evaluated dataset setting.

**Figure 10:**
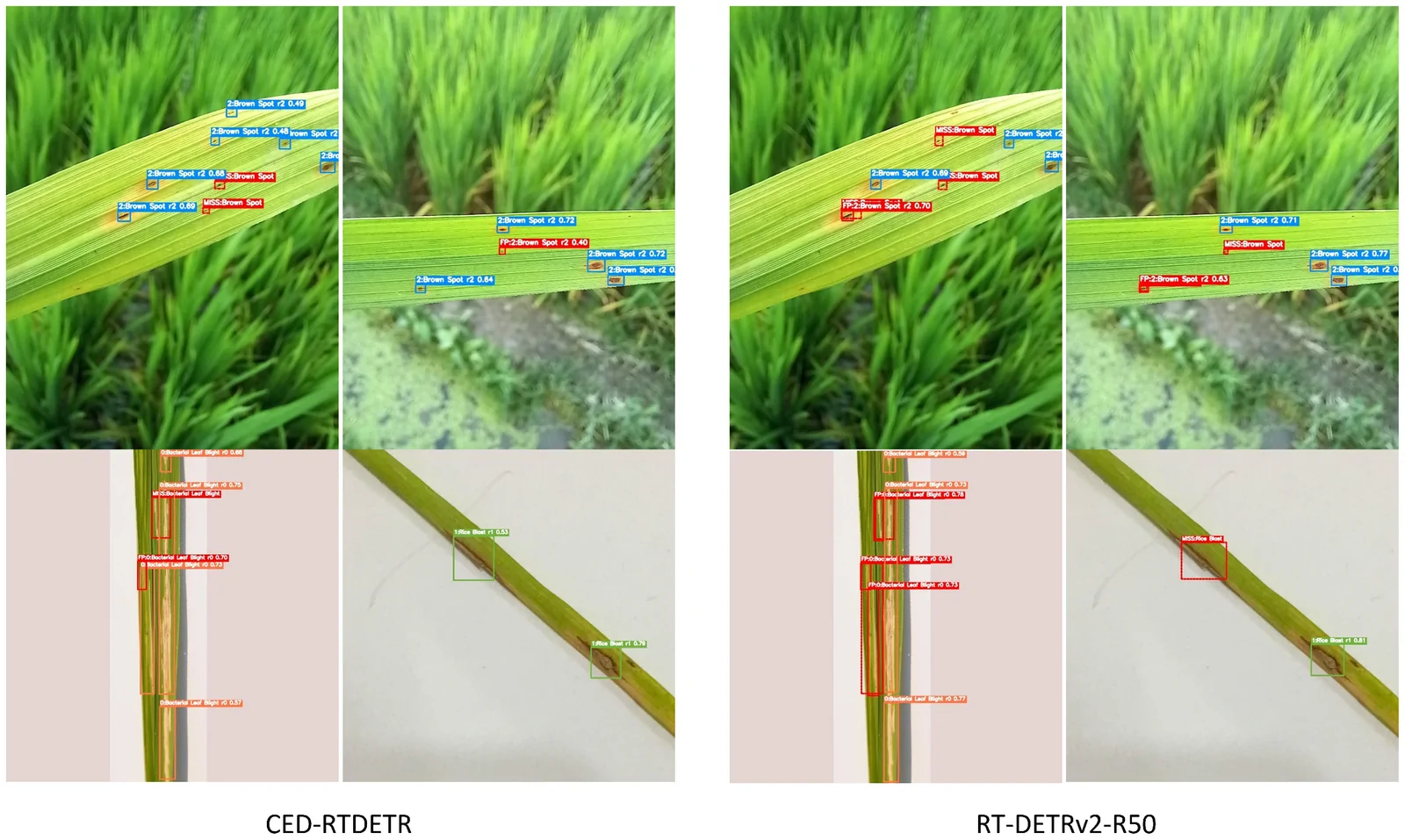
Qualitative comparison of representative missed detections and false positives.

To further provide a more balanced qualitative assessment, representative missed-detection and false-positive cases were compared between CED-RTDETR and the RT-DETRv2-R50 baseline, as shown in **Figure 10**. In this figure, normal predicted boxes are shown in category-specific colors, whereas red boxes indicate false positives or missed detections.

As shown in **Figure 10**, CED-RTDETR achieves more reliable qualitative detection results than the RT-DETRv2-R50 baseline. For small and scattered brown spot lesions, the improved model detects more lesion regions along the leaf surface and reduces the number of missed detections observed in the baseline. This indicates that the contour-aware backbone and multi-scale evidence fusion neck improve the model’s sensitivity to small lesion textures and weak boundary cues. For elongated bacterial leaf blight lesions, CED-RTDETR provides more continuous localization of stripe-like diseased regions, suggesting that direction-aware spatial mixing and contour-guided enhancement are beneficial for preserving weak and slender lesion structures. In the rice blast example, the improved model also detects weak-contrast lesion regions that are missed by the baseline, further indicating improved lesion localization stability. However, several failure cases remain. False positives may still occur when leaf veins, shadows, or local background textures show color and shape patterns similar to lesion regions. Missed detections may also appear for extremely small, blurred, or low-contrast lesions. In addition, when multiple tiny lesions are densely distributed or when lesion boundaries are weak, the predicted boxes may be slightly shifted or fail to fully align with the ground-truth boxes. These observations suggest that although CED-RTDETR improves the qualitative detection stability for small, elongated, and weak-boundary lesions, high-precision localization under dense and ambiguous lesion patterns remains challenging. This is also consistent with the relatively lower AP75 compared with AP50, indicating that stricter localization quality remains an important direction for future improvement.

## 5 Conclusion

To address the challenges of large lesion-scale variations, weak boundary cues, and confusion among visually similar rice disease categories, this study proposes CED-RTDETR, an improved rice leaf disease detection model based on RT-DETRv2-R50. In the backbone, LCGA-Backbone combines the InceptionDWConv2d-based DIDM strategy with CEGA Blocks after the C3, C4, and C5 stages, enhancing directional lesion textures, weak boundaries, and contextual semantics while keeping the additional spatial modeling cost controllable. In the neck, MEIF-Neck introduces MDEI and SE-RepNCSPELAN to perform cross-scale evidence interaction, salient-region selection, lightweight reconstruction, and background-interference suppression. In the decoder, DAD-DA decomposes deformable attention sampling offsets into direction residuals and radius gains, and introduces aspect-ratio-aware geometric compression-restoration to improve sampling stability for slender and irregular lesion regions.

On the constructed rice leaf disease dataset, CED-RTDETR achieves AP, AP50, and AP75 values of 29.5%, 75.2%, and 17.6%, respectively, outperforming RT-DETRv2-R50 by 4.1, 4.9, and 3.8 percentage points. On the public Rice Disease Dataset, the proposed method further achieves AP, AP50, and AP75 values of 34.1%, 77.1%, and 23.2%, respectively, with corresponding improvements of 4.3, 5.6, and 3.4 percentage points over RT-DETRv2-R50. These results show that CED-RTDETR achieves consistent overall performance gains across the constructed dataset and the public benchmark dataset, indicating improved lesion response intensity, target recall, and bounding-box localization quality under the evaluated dataset settings.

It should be noted that the present study focuses on bounding-box-based lesion localization rather than complete disease assessment. Therefore, the proposed method can identify disease categories and locate visible lesion regions, but it does not directly estimate lesion area, disease severity, infection stage, or field-scale disease distribution. These tasks usually require more detailed annotation information, such as pixel-level lesion masks, severity grades, temporal disease progression records, and field-level sampling data. In addition, although the proposed method shows improved detection performance on the evaluated datasets, several limitations remain. First, the scale and source diversity of the dataset are still limited, and some public images are subject to data-use and redistribution restrictions. Second, the AP75 value remains relatively low compared with AP50, indicating that high-precision lesion localization is still challenging for small, scattered, weak-boundary, and irregular lesions. Third, the current experiments are mainly dataset-level evaluations, and independent external validation under different imaging devices, rice varieties, growth stages, field environments, and disease occurrence conditions has not yet been fully conducted. Finally, practical deployment still requires further consideration of image acquisition variability, real-time inference constraints on edge devices, and integration with existing crop management workflows.

Future work will therefore proceed in several directions. More independently collected field images will be added to expand the dataset across diverse illumination conditions, imaging distances, device types, rice varieties, growth stages, and disease backgrounds. Cross-source and field-level validation will be conducted to evaluate the generalization ability of the model more comprehensively. In addition, pixel-level lesion segmentation, lesion area calculation, disease severity estimation, and infection-stage recognition will be further explored to extend the current bounding-box detection framework toward more complete rice disease assessment. For practical use, future work will also investigate lightweight deployment, real-time inference optimization, and the integration of detection results with field scouting, disease recording, and crop management decision-support workflows.

## Data Availability

The original images used in this study were selected from publicly available rice disease image resources and the Roboflow community. The processed dataset generated in this study, including manually revised annotations, dataset splits, and derived augmented data, is not publicly available because some original images may be subject to third-party redistribution restrictions and the curated dataset is being used for ongoing research. Access requests should be directed to the corresponding author and will be considered subject to reasonable academic use and applicable data-use restrictions. Requests to access these datasets should be directed to Chunxi Zhao, zcx@jlau.edu.cn.
